# Sponge bioerosion on changing reefs: ocean warming poses physiological constraints to the success of a photosymbiotic excavating sponge

**DOI:** 10.1038/s41598-017-10947-1

**Published:** 2017-09-06

**Authors:** Michelle Achlatis, Rene M. van der Zande, Christine H. L. Schönberg, James K. H. Fang, Ove Hoegh-Guldberg, Sophie Dove

**Affiliations:** 1 The University of Queensland, School of Biological Sciences, Coral Reef Ecosystems Laboratory, St. Lucia, QLD 4072 Australia; 2 The University of Queensland, Australian Research Council Centre of Excellence for Coral Reef Studies, St. Lucia, QLD 4072 Australia; 3 The University of Queensland, Global Change Institute, St. Lucia, QLD 4072 Australia; 4 The University of Western Australia, School of Earth and Environment and Oceans Institute, Crawley, WA 6009 Australia; 5 The Hong Kong Polytechnic University, Department of Applied Biology and Chemical Technology, Hung Hom, Kowloon Hong Kong

## Abstract

Excavating sponges are prominent bioeroders on coral reefs that in comparison to other benthic organisms may suffer less or may even benefit from warmer, more acidic and more eutrophic waters. Here, the photosymbiotic excavating sponge *Cliona orientalis* from the Great Barrier Reef was subjected to a prolonged simulation of both global and local environmental change: future seawater temperature, partial pressure of carbon dioxide (as for 2100 summer conditions under “business-as-usual” emissions), and diet supplementation with particulate organics. The individual and combined effects of the three factors on the bioerosion rates, metabolic oxygen and carbon flux, biomass change and survival of the sponge were monitored over the height of summer. Diet supplementation accelerated bioerosion rates. Acidification alone did not have a strong effect on total bioerosion or survival rates, yet it co-occurred with reduced heterotrophy. Warming above 30 °C (+2.7 °C above the local maximum monthly mean) caused extensive bleaching, lower bioerosion, and prevailing mortality, overriding the other factors and suggesting a strong metabolic dependence of the sponge on its resident symbionts. The growth, bioerosion capacity and likelihood of survival of *C*. *orientalis* and similar photosymbiotic excavating sponges could be substantially reduced rather than increased on end-of-the-century reefs under “business-as-usual” emission profiles.

## Introduction

To date approximately 30% of the anthropogenic carbon dioxide (CO_2_) emissions have been absorbed by the oceans^1^. Since the beginning of the Industrial Revolution, ocean pH has decreased by 0.1 units^2^. In combination with ocean acidification, ocean warming caused by the CO_2_-driven enhancement of the greenhouse effect, and eutrophication are threatening the distribution and abundance of coral reefs worldwide^3–5^. Projections conclude that these changes will reduce calcium carbonate (CaCO_3_) accretion on reefs due to increased mortality and decreased calcification potential of reef-building organisms^6–8^. Compared to calcification, responses of decalcification and especially biological erosion (bioerosion) to environmental change remain less well studied^9^, even though bioerosion is of equal significance to the carbonate balance on coral reefs^10, 11^.

Bioeroding taxa on coral reefs include internal bioeroders that excavate and inhabit CaCO_3_ materials (e.g. certain poriferan, molluscan, annelid, algal, fungal and cyanobacterial genera) and external bioeroders (e.g. certain echinoderm, crustacean, molluscan and fish genera)^12^. As it is predominantly the internal bioeroders that employ chemical means to rework the substrate, and as they are mostly sessile, changes to seawater chemistry may be directly reflected in their bioerosion capacity^11^. Attention on internal bioerosion has focused more specifically on coral-excavating sponges^9^, which often account for 40–70% and up to >90% of macroborer activity on coral reefs^10^ (references therein). Excavating sponges influence seawater carbonate cycling and they play important ecological roles by breaking down and sculpting the reef framework, thereby changing the heterogeneity and availability of space^10^. In contrast to many calcifiers, the abundance, activity and competitive vigour of certain excavating sponges has been observed to increase on perturbed reefs (e.g. refs 13–16).

As excavating sponges are demosponges that have a siliceous skeleton, their skeletogenesis is unlikely to be as strongly impacted by carbonate saturation changes as that of calcifiers^17^. However, ocean warming, acidification and eutrophication may still affect these sponges for several reasons. Sponge bioerosion proceeds through chemical etching of CaCO_3_ chips, which purportedly involves acid regulation, followed by mechanical removal of the chips from the substrate^18, 19^. The energetic cost of chemical bioerosion may be reduced in more acidified oceans, as the CaCO_3_ dissolution threshold will be more easily met^20, 21^. As a result, bioerosion may be enhanced on future reefs^22^, as has been suggested after observing bioerosion patterns in CaCO_3_ materials from naturally low-pH waters^23–26^. Some dominant bioeroding sponges are aggressive space competitors capable of overgrowing living corals^27, 28^, and reduced competitive pressure caused by increased weakness and mortality of corals may further elevate their abundances^13, 14, 29, 30^. Moreover, bioeroding sponges are filter feeders with an efficient pumping system^31, 32^ and they have been observed to thrive in eutrophic waters^33–35^. Eutrophication may result in greater access to food and thereby increase energetic availability, which may not only affect sponge abundances and growth, but may also increase their bioerosion rates^36^.

Apart from their heterotrophic filter feeding, certain bioeroding sponges also benefit from photoautotrophic inputs provided by symbiotic dinoflagellates of the genus *Symbiodinium* ^37^. The *Cliona viridis* species complex consists of such species, which are very competitive and destructive to the CaCO_3_ framework^10^. Presumably, this is due to the symbiosis providing greater access to energy and hence promoting greater sponge growth, survival and bioerosion^38–40^. In comparison to corals, these sponges are also thought to be relatively resilient to bleaching^10^, which suggests that they are well-positioned to dominate newly available space should corals decline^13–15^.

The potential of increased bioerosion by excavating sponges in changing environments implies a growing threat to the three-dimensional framework of future reefs and the organisms that inhabit them^41^. However, the physiological limits to the enhanced performance of excavating sponges remain to be explored. Research on photosymbiotic bioeroding sponges under experimentally elevated partial pressure of CO_2_ (*p*CO_2_) displayed only little adverse response or accelerated bioerosion (reviewed in ref. 11). Experiments observing effects of elevated temperature have either not shown a strong response^42, 43^, or led to bleaching^44^ (also BD Ramsby, pers. comm.) or partial necrosis or mortality^45^. Combined exposure of the Indo-Pacific photosymbiotic sponge *Cliona orientalis* to temperature and *p*CO_2_ anomalies under two future scenarios over eight weeks (Austral spring to summer transition) dramatically enhanced both the growth and bioerosion of the sponge^44^. However, under spring business-as-usual conditions *C*. *orientalis* bleached and showed energetic deficiencies^44, 46^ that are likely to lead to mortality in the longer term over summer (JKH Fang, pers. comm.).

The current study assessed the capacity of *C*. *orientalis* to erode and survive over the summer on future reefs through a 10-week simulation of both independent and concurrent warming and acidification predicted for the year 2100. To explore the influence of local nutrient availability on the outcome of globally changing climate conditions, supplementation of the diet of the sponges with nitrogen-rich particulate organics was also included as a factor. The diurnally and seasonally variable simulation was based on a “business-as-usual” greenhouse gas concentration trajectory called the Representative Concentration Pathway 8.5 (RCP8.5)^1^. As opposed to previous experiments, the individual and combined effects of temperature, *p*CO_2_ and diet supplementation on bioerosion rates, biomass, oxygen flux, carbon flux and survivorship were studied in an orthogonal design. Such designs permit the unravelling of independent effects, which is crucial to fully understand the physiological mechanisms underpinning an organism’s response. The key questions of the current study were: (1) Do simulated warming and/or acidification and/or diet supplementation accelerate or decelerate bioerosion rates and growth of *C*. *orientalis*? (2) What changes to the carbon budget of the sponge drive the observed responses? (3) Which of the three factors or which of the combinations is most likely to impact the sponge’s survival in future oceans under RCP8.5 emissions?

## Methods

### Experimental design and sample collection

**Figure 1:**
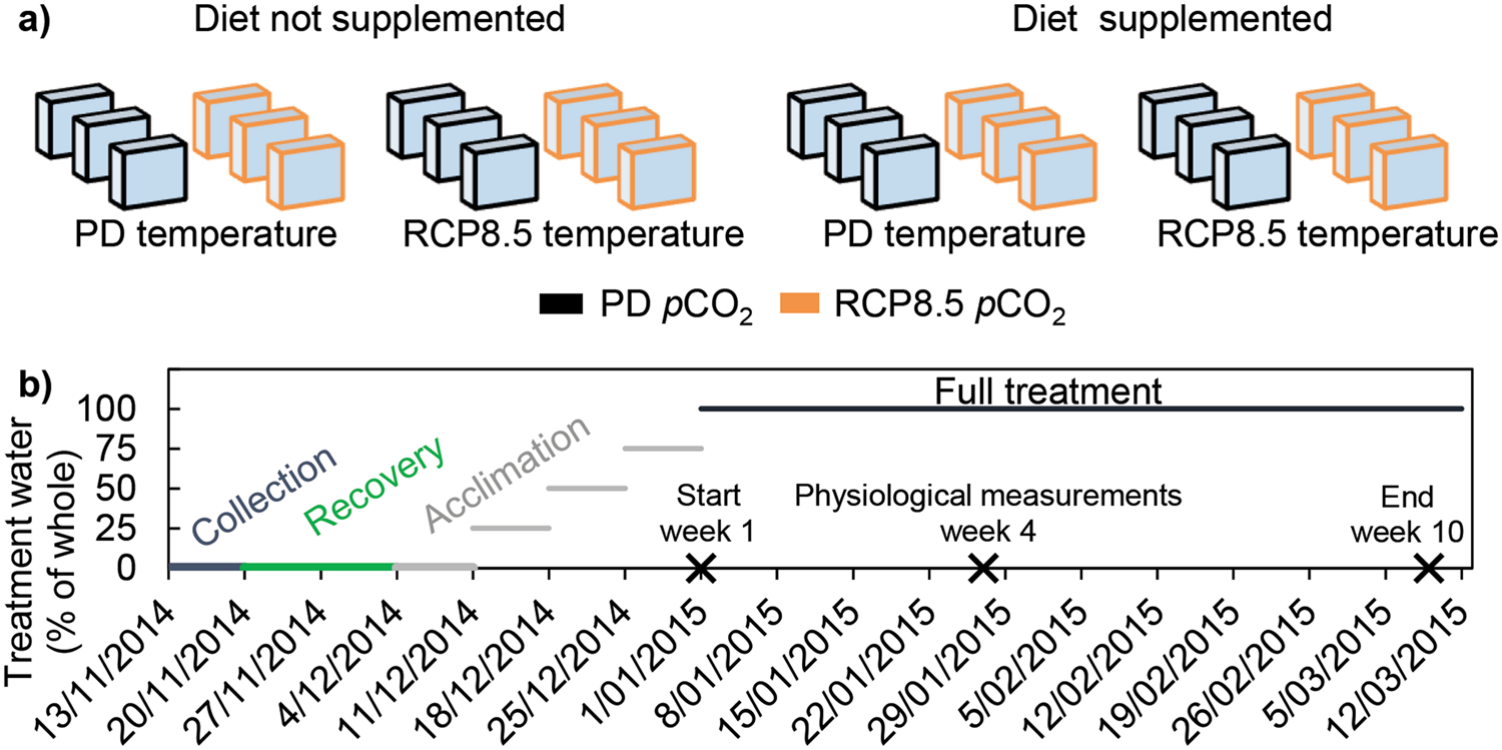
Schematic illustration of experimental design and timeline. (**a**) The orthogonal setup of the experiment produced 8 different combinations using three factors (temperature, *p*CO_2_ and diet, with 2 levels each) and 3 replicate aquaria for each combination (i.e. 2 temperatures × 2 *p*CO_2_ levels × 2 diets × 3 replicate aquaria = 24 aquaria in total). These aquaria were randomly assorted on the experimental table, so as not to bias the amount of light that specific treatments received. A total of 96 sponge cores and 96 controls cores from different sponge-invaded dead coral substrates were distributed across the aquaria (n = 12 per treatment which equals to n = 4 per aquarium). The cores were rotated in preassigned groups between replicate aquaria of the same treatment every 4th day to minimize localized variations in light intensity and other positional effects^59–61^. All aquaria were equipped with temperature loggers, light loggers and small wave makers and covered with light filters (Lee Filters #131) to mimic the light spectrum of the collection depth^88^ (**b**) After collection, the sponge cores recovered for 2 weeks to allow full healing before being exposed to a stepwise acclimation to the experimental treatments (progressive weekly steps of 0, 25, 50, 75 and 100% treatment water). Full treatment (100% treatment water) was reached on the 1^st^ of January (start) and maintained until the 9^th^ of March 2015 (end). Physiological measurements were performed with a subset of the sponges in the 4^th^ week after the onset of full treatment conditions (“week 4” of the experiment).

Experiments were conducted at the Heron Island Research Station on the southern Great Barrier Reef over the Austral summer 2014–2015 using the ocean warming and acidification simulation system^47, 48^ (see also Supplementary Information). This system allows computer-controlled manipulation of seawater chemistry while mimicking natural diurnal and seasonal variability of the reef. Four different flow-through scenarios were produced in 4 mixing sumps under a fully orthogonal combination of temperature and *p*CO_2_ according to the greenhouse gas concentration trajectory RCP8.5^1^ (Fig. 1a and Figs S1 and S2):

- Baseline simulation, reproducing conditions at the reference site, with present-day (PD) levels of both temperature and *p*CO_2_.
- Elevated *p*CO_2_ only, with RCP8.5 *p*CO_2_ levels (set at 572 ± 11 μatm above PD) while maintaining PD temperature levels.
- Elevated temperature only, with RCP8.5 temperature (3.5 °C above PD) while maintaining PD *p*CO_2_ levels.
- Concurrent elevation of temperature and *p*CO_2_ to RCP8.5 levels.

Dietary supplementation was superimposed on these scenarios in orthogonal setup, dividing the experiment into supplemented and unsupplemented treatments, with three replicate 40 L experimental aquaria ascribed to each treatment (24 aquaria in total, see text below and Fig. 1a). Seawater supplying the aquaria contained dissolved organics and picoplankton cells up to 10 μm diameter that comprise the main part of sponges’ diets, including clionaids^31, 49, 50^. The water flow to the aquaria (1 L/min in each aquarium) was interrupted for one hour daily and diet-supplemented aquaria received a dose of protein-rich microalgal mix to a final concentration of 25 μL L^−1^ (non-viable, 9% algal biomass, N-rich High Pro, Reed Mariculture, Campbell, USA). Sunset was chosen as the feeding time, since it follows the period of maximum mucus production by corals triggering bacterial enrichment and increased food supply for reef organisms^51, 52^. The sponges in the present experiment were still pumping actively at sunset (confirmed with fluorescent dye) and their oscula were open.

The experiment was performed with *Cliona orientalis* Thiele, 1900 in encrusting “beta” morphology^10, 11^. Apart from *Symbiodinium*, *C*. *orientalis* hosts a low abundance of other microorganisms^53^ (such as *Alpha* and *Gammaproteobacteria*)^54^. In the current study, treatment responses were addressed at the holobiont level. Samples were collected from *C*. *orientalis* individuals inhabiting dead coral substrates at 5 m depth at Harry’s Bommie (151.9357°E, 23.4675°S), Heron Island in November 2014. Sponge spicules were examined to confirm the species identity^55^. The sponges were cored to produce standardized cylinders of 35 mm diameter. Each core was then horizontally divided into 15 mm slices. The upper slice contained the sponge tissue plus a ca. 3 mm-thin underlying disc of non-infested CaCO_3_ to allow downward growth and expansion (sponge core), whereas the lower slice consisted of entirely sponge-free CaCO_3_ substrate (control core). Control cores were bleached for 6 h in 12.5% sodium hypochlorite to remove organic matter, thoroughly washed in distilled water and then reconditioned in running seawater for at least 48 h. Control cores served throughout the experiment to quantify background weight loss due to passive CaCO_3_ dissolution, abrasion from cleaning and bioerosion by re-colonizing microborers. To eliminate variation in bioerosion rates caused by substrate properties^56^, only material sampled from six sponges excavating CaCO_3_ of similar bulk density was selected (measured as outlined in Supplementary Information and Fig. S3). The sponge-free CaCO_3_ of the selected individuals had a higher initial bulk density (2.30 ± 0.02 g/cm^3^, mean ± SEM) compared to the massive *Porites* sp. (<1.6 g/cm^3^, e.g. ref. 57) eroded by *C*. *orientalis* in previous experiments^20, 44^.

The sponge cores (n = 12 per treatment) and control cores (n = 12 per treatment) from the six genotypes were labelled and randomly distributed^58^ across the 24 aquaria (4 sponge cores and 4 control cores per aquarium), with each of the 8 treatments receiving two cores of each genotype where possible. The contents of each aquarium were then assigned a group label (A through X). After collection, the sponge cores recovered for 2 weeks to allow full healing (open oscula on all core surfaces) before being exposed to a stepwise acclimation to the experimental treatments (progressive weekly steps of 0, 25, 50, 75 and 100% treatment water mixed with ambient seawater). Subsequently, full treatment conditions were maintained for 10 weeks (Fig. 1b). During the experiment, the preassigned groups of cores were rotated every 4th day between replicate aquaria (after water renewal) of the same treatment to minimize localized variations in light intensity and other positional effects^59–61^. Upstream temperature and *p*CO_2_ were continuously monitored in the four mixing sumps, while downstream temperature and pH were continuously monitored in the experimental tanks, with additional weekly pH measurements made inside each of the 24 aquaria (see Supplementary Methods for details). The use of a single sump per experimental treatment is technically a pseudo-replicated design^62^. The flow rate and dimensions of the dark sump however significantly limit the potential for confounding effects (see Supplementary Methods). Replicating sumps would necessitate a counterproductive reduction in sump size, significant increases to the energy budget on an offshore island, and would prove to be impractical for an experiment that aims to deliver *in situ* diurnal fluctuations for multiple factors in reef water of high quality.

Four weeks after the onset of full treatment conditions (hereafter referred to as “week 4”, Fig. 1b), physiological measurements were performed with a subset of the specimens, which were subsequently sacrificed (n = 7 out of 12 per treatment). At this point in time the top surfaces of all cores were still entirely covered by sponge tissue and oscula were open. The remaining specimens (n = 5) were retained until the end of the summer period. During the experiment, aerial exposure of the sponges was prevented and epibionts were removed regularly using a pair of forceps and/or a soft brush, taking care not to damage the sponge tissue.

### Bleaching and mortality

**Figure 2:**
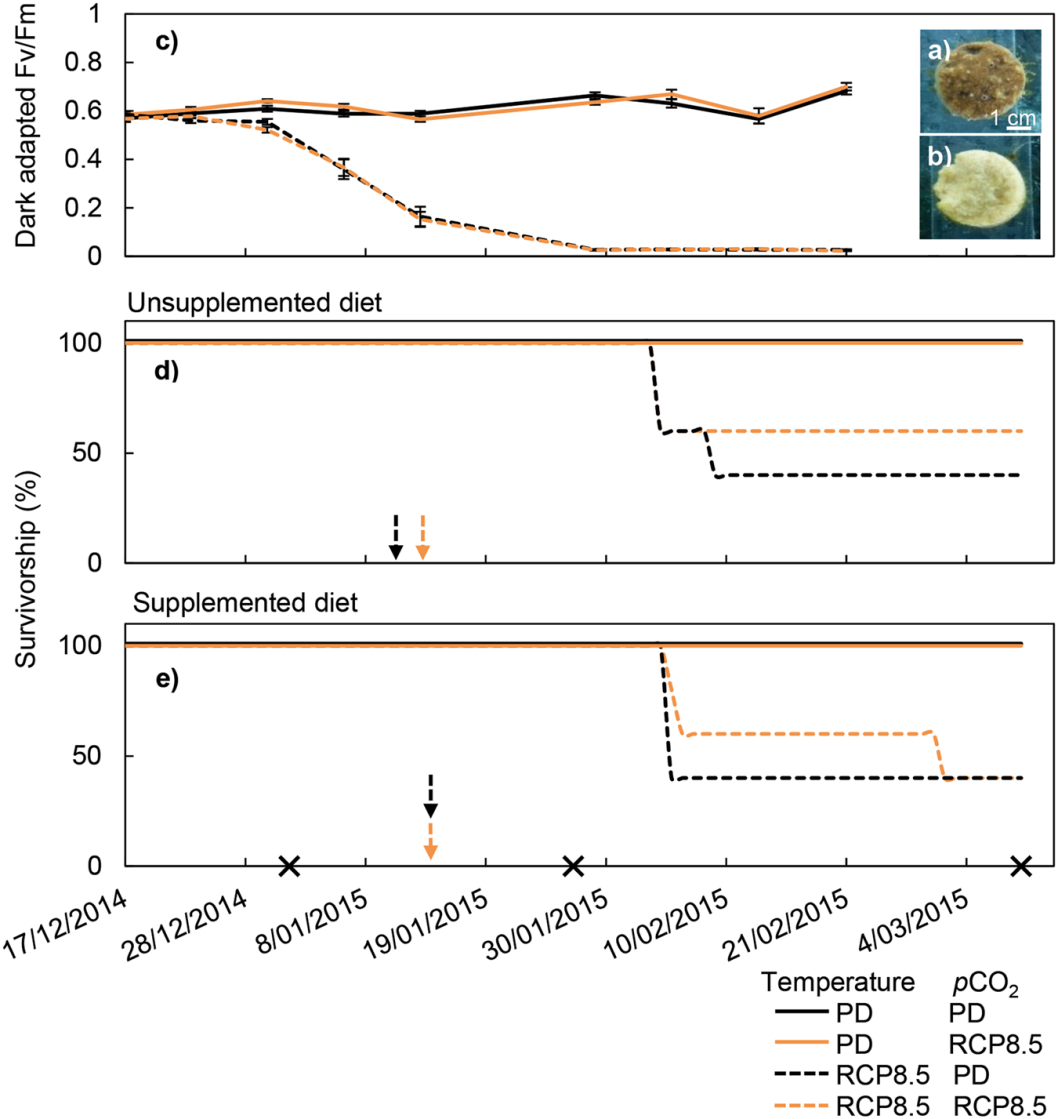
Photophysiology and survival of the *Cliona orientalis* holobiont under independent and concurrent simulation of warming and acidification from present-day (PD) summer conditions to Representative Concentration Pathway 8.5 (RCP8.5) conditions of the year 2100. Insets (**a**) and (**b**) show typical colours of healthy (brown) and bleached (pale yellow) sponges as observed in the treatments with PD versus RCP8.5 temperature levels respectively. (**c**) Dark-acclimated Fv/Fm of *Symbiodinium* in the sponge cores over the course of the experiment reflects the bleaching observations (mean ± SEM). Data are averaged across diet, since diet did not influence Fv/Fm ratios. Survival of *C*. *orientalis* decreased over the summer in all heated treatments, both for treatments with unsupplemented (**d**) and supplemented diets (**e**). Only complete mortality was considered for this comparison. Crosses represent the arrival at full treatment conditions, the 4^th^ week thereafter when physiological measurements were performed and the end of the experiment respectively. Arrows indicate the points in time when 100% bleaching of sponge cores in heated treatments were reached.

Bleaching, partial and complete mortality of the sponge cores were scored throughout the experiment. Bleaching was assessed by the colour change of the sponges from dark brown to pale yellow (Fig. 2a,b) and confirmed upon dissection to eliminate the possibility that symbionts were relocated deeper inside the core^63^. As a measure of photoinactivation during bleaching, the maximum potential quantum yield of photosystem II (Fv/Fm) of the sponge symbionts was measured once a week at 20:00 h using pulse amplitude modulated (PAM) chlorophyll fluorometry^64^ (dark-acclimated; Diving-PAM, Walz, Effeltrich, Germany). The PAM measurements (in combination with the oxygen flux assay, see below) further confirmed the observed bleaching^46, 65^. Towards the end of the experiment, some sponge cores (only in the treatments where diet was not supplemented) experienced partial mortality, defined here as the loss of sponge tissue from areas of the core, partially exposing the CaCO_3_ substrate but without the appearance of black patches. The assessment of complete mortality was based on blackening of all sponge tissue, decaying odour and absence of oscula^45^.

### Bioerosion rates

To measure total sponge bioerosion over time, all sponge and control cores were buoyant-weighed^66^ at the onset of the full-treatments, at week 4 and at the end of the experiment. The decrease in the buoyant mass (BM) of the sponge cores was calibrated for seawater density and corrected for residual mass loss using the control cores. Changes in BM were converted to changes in CaCO_3_ mass of the cores (see Supplementary Information). The upper surface areas of sacrificed sponge explants were estimated using a standard aluminium foil method^67^ and total bioerosion rates were expressed as mg CaCO_3_ cm^−2^ day^−1^ in reference to this surface.

### Metabolic oxygen and carbon flux

Photosynthetic and respiratory activities of the sponge cores (n = 7 replicates per treatment) were assessed at week 4 of the experiment, using an OXY-10 oxygen meter (PreSens, Regensburg, Germany) as described in detail previously^46^. Sponges were 30-min dark-acclimated before being placed -without exposing them to air- inside sealable chambers (250 ml) filled with respective scenario seawater (filtered to 0.45 μm to remove most microorganisms from the seawater), and non-viable algal mix was added to supplement half of the treatments to the same per sponge concentration as during daily supplementation of the experimental aquaria. The sponges were subjected to a cycle of 30 min darkness followed by 20 min light at 450 μmol quanta m^−2^ s^−1^ (Aqua Medic Ocean lights, Bissendorf, Germany), with oxygen levels logged every 15 s. Dark respiration (R_dark_) and maximum net photosynthesis (max P_net_ at saturating light intensity)^65^ were quantified based on the oxygen depletion or evolution (μM O_2_ cm^−2^ h^−1^) during the dark and light phase of the cycle respectively. R_dark_ reflects changes in holobiont metabolism as well as pumping rates (not measured here). The same procedure was performed with the control cores, which generated minimal non-sponge metabolic rates that were used to correct the sponge results. Corrected photosynthetic values were ascribed to *Symbiodinium*. The hourly respiratory and net photosynthetic rates were scaled up to daily rates and converted into carbon equivalents (see Supplementary Information).

To measure the uptake or excretion of particulate and dissolved organic carbon by the sponges (POC and DOC respectively), incubations in confined seawater (5 L) were performed^46^. Dietary supplementation of the incubated sponges took place in the same manner as daily in the experimental aquaria. Seawater samples were collected and analysed as detailed in the Supplementary Information. Uptake rates of DOC and POC were expressed as mg C cm^−2^ day^−1^ and added to the carbon flux that was calculated from photosynthesis and respiration. In that manner, the daily net carbon surplus (C_net_) available to the sponge holobiont was estimated.

### Biomass

After completion of the week 4 physiological measurements, analysed sponge and control cores were snap-frozen in liquid nitrogen and stored at −80 °C. The sponge cores were later vertically divided into 4 quarters (A, B, C and D), three of which were subjected to different analyses. To quantify total organic mass of the core, spicular mass of *C*. *orientalis* and CaCO_3_ mass (M_CaCO3_), a loss after combustion method was performed on quarter A with correction factors applied^66^; to quantify organic mass of other endo/epilithic organisms that could inhabit the sponge holobiont, an acid decalcification method was conducted with quarter B^66^. The organic mass of *C*. *orientalis* was estimated by subtracting the organic mass of other organisms from the total organic mass. All biomass data were standardized to CaCO_3_ mass of the whole core (mg_(biomass)_/g_(core)_). Quarter C was used to quantify the bulk density of the sponge core (Supplementary Information and Fig. S3).

### Data analysis

Preliminary analysis did not detect group-specific effects for any of the variables and therefore replicate groups of each treatment were pooled^68^. No single genotype or group of genotypes deviated from the others in a way that would substantially bias the analysis (Fig. S4). Dependent variables were analysed using a factorial, fully crossed analysis of variance (ANOVA) with three categorical factors having two levels each, resulting in a 2 × 2 × 2 matrix: temperature (PD and RCP8.5), *p*CO_2_ (PD and RCP8.5), diet (supplemented and unsupplemented; Fig. 1a). Datasets were tested for normality (Shapiro-Wilk test) and homogeneity of variances (Levene’s test). Log or square root transformations were applied if normality was violated, and heteroscedastic datasets were assesed at a reduced alpha level of 0.01^68^ (Table S4). Main effects were reported when there were no significant three-way or two-way interactive effects. Differences in the case of significant three-way interactions were explored using simple two-way interactions at each level of the third factor whilst using the error term (sum of squares and degrees of freedom) of the three-way ANOVA^69^. If such simple two-way interactions were significant, pairwise comparisons with Bonferroni adjustments (to reduce type I errors) were performed to determine the effect of a factor at each level of the other factor^69^. Chlorophyll fluorescence data were analysed with a three-way repeated measures ANOVA examining the effects and interactions of the categorical factors over time. Partial and complete mortality of the sponge cores were assessed at the end of the experiment with a two-way ANOVA and a two-proportions z-test. Statistical analyses were evaluated at a 0.05 level of significance (unless otherwise stated) and were performed using SPSS Statistics software (IBM, New York).

### Data availability

The datasets generated and analysed during the current study are available from the corresponding author on reasonable request.

## Results

### Treatment conditions

Mean *p*CO_2_ levels across the experiment were 493 ± 20 μatm (mean ± SEM hereafter, unless otherwise specified) for treatments with PD levels and 982 ± 17 μatm for treatments with RCP8.5 levels, thus approximately denoting a doubling from PD to RCP8.5. Peak temperature levels of 28.3 ± 0.1 °C for the PD scenarios and 31.6 ± 0.1 °C for the elevated scenarios [+1 and +4.3 °C above the maximum monthly mean (MMM) respectively^70^] were reached four weeks after the onset of full treatment conditions (Fig. S1). Details of the experimental conditions are given in the Supplementary Information (Figs S1 and S2 and Tables S1 and S2).

### Bleaching of sponge cores

Sponges in the heated treatments began to pale 24 days after the onset of acclimation, when midday temperature was 29.4 ± 0.06 °C (MMM + 2.1 °C) and midday *p*CO_2_ was 452 μatm in the low *p*CO_2_ treatment and 903 μatm in the high *p*CO_2_ treatment. By the onset of full treatment conditions, approximately one third of all the sponges exposed to simulated warming were visibly bleached (Fig. 2a,b). Bleaching of 100% of the sponge population in each heated treatment was reached after approximately the same number of full treatment days, regardless of diet (arrows in Fig. 2d,e). In line with the bleaching observations, maximum potential quantum yield of fluorescence for photosystem II of *Symbiodinium* decreased significantly in all heated treatments (F = 53.76, p < 0.001; Fig. 2c). Throughout the experiment, bleaching occurred only in the heated treatments.

### Bioerosion rates

**Figure 3:**
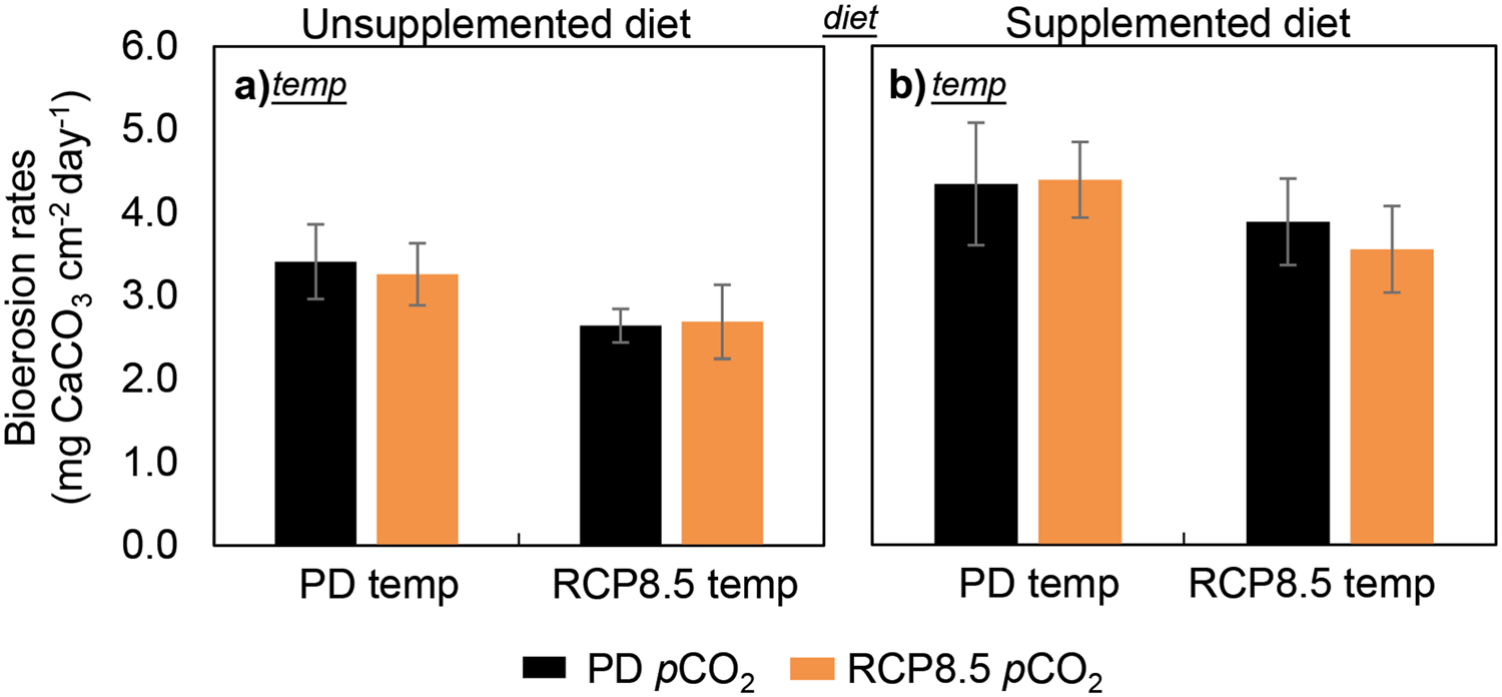
Total bioerosion rates of *Cliona orientalis* at Heron Island on the southern Great Barrier Reef receiving an unsupplemented (**a**) and supplemented diet (**b**) under independent and concurrent simulation of warming and acidification from present-day (PD) summer levels to Representative Concentration Pathway 8.5 (RCP8.5) levels of the year 2100 (mean ± SEM). The rates represent the loss of CaCO_3_ measured as buoyant mass change over the first 4 weeks of full treatment conditions. The designations *diet* or *temp* indicate significant main effects of diet supplementation or temperature respectively. Buoyant mass at the end of the experiment (week 10) is not presented here due to prevalent mortality in the heated treatments from week 6 onwards.

Total sponge bioerosion corrected for control values ranged between 2.64 to 4.39 mg CaCO_3_ cm^−2^ day^−1^ over the first 4 weeks of full treatment and was on average 35% higher for sponges with supplemented diets compared to unsupplemented sponges (F_(1,48)_ = 8.07, p = 0.007; Fig. 3a,b; Table S4). Simulated warming lowered total bioerosion rates by 20% (F_(1,48)_ = 4.54, p = 0.038), whereas acidification on its own did not have a strong effect on total bioerosion. Rates of weight loss in the sponge-free control cores were an order of magnitude lower than sponge bioerosion rates (0.28–0.59 mg CaCO_3_ cm^−2^ day^−1^) and did not differ between the treatments.

The bulk densities of the sponge cores at week 4 ranged from 1.67 to 1.82 g cm^−3^ across the different treatments with the average porosity being 35.90 ± 0.94%. The lowest densities and highest porosities were found in the treatments with both PD temperature and PD *p*CO_2_, yet these differences were not significant.

### Metabolic oxygen and carbon flux

**Figure 4:**
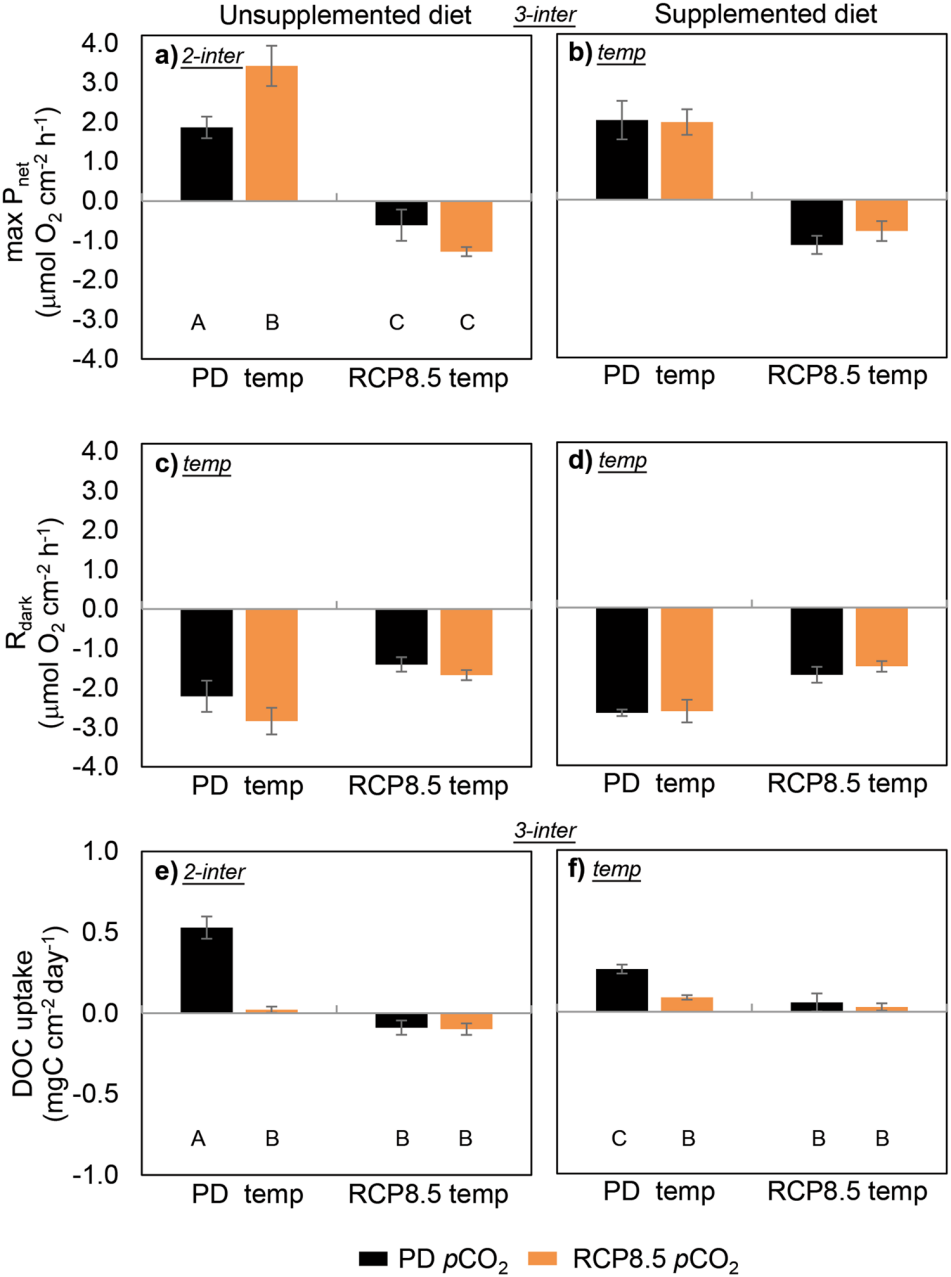
Maximum net photosynthesis (max P_net_), dark respiration (R_dark_) and dissolved organic carbon (DOC) uptake of *Cliona orientalis* at Heron Island on the southern Great Barrier Reef receiving an unsupplemented (**a**,**c** and **e** respectively) and supplemented diet (**b**,**d** and **f** respectively) under independent and concurrent simulation of warming and acidification from present-day (PD) summer levels to Representative Concentration Pathway 8.5 (RCP8.5) levels of the year 2100 (mean ± SEM). All parameters were measured 4 weeks after the onset of full treatment conditions. Max P_net_ and R_dark_ rates represent hourly rates of oxygen evolution and depletion, whereas DOC uptake was measured over a 24 h period. The designations *inter*, *temp*, or p *CO*_*2*_ indicate significant 3-way or 2-way interactive effects as specified, or (simple) main effects for temperature or *p*CO_2_ respectively within each panel. When interactive effects are present, the capitalized letters beneath the bars indicate statistical differences. When standardized to sponge biomass, reported R_dark_ rates (**c** and **d**) range between 14.46 ± 1.22 and 24.60 ± 1.49 μmol O_2_ g^−1^ h^−1^ (mean ± SEM).

Photosynthesis in week 4 of the experiment exceeded respiration rates only in the absence of simulated warming where the sponges did not bleach (max P_net_, Fig. 4a,b and Table S4). When the diet was not supplemented, acidification enhanced photosynthesis by 80% (F_(1,48)_ = 5.96, p = 0.018), but this effect was absent when the diet was supplemented. Warming, acidification and diet interactively drove the response of photosynthesis (3-way interaction, F_(1,48)_ = 6.48, p = 0.014). Dark respiration (R_dark_, Fig. 4c,d) of the sponge cores was affected only by temperature, with sponges under RCP8.5 temperature respiring at a slower rate (F_(1,48)_ = 36.44, p = 0.00).

Diet supplementation caused increased uptake of dissolved organic carbon (DOC, Fig. 4e,f and Table S4), but this food source appeared to be only half consumed when the sponges were under PD temperature and *p*CO_2_ levels (F_(1,48)_ = 26.06, p < 0.001, Fig. 4e). Warming led to a decrease in DOC uptake (F_(1,48)_ = 141.76, p < 0.001 and F_(1,48)_ = 13.09, p = 0.001 for unsupplemented and supplemented treatments respectively, Fig. 4e,f). Acidification lowered uptake rates at PD temperatures, regardless of diet (95% decrease, F_(1,48)_ = 95.43, p < 0.001 and 65% decrease, F_(1,48)_ = 11.41, p = 0.001 for unsupplemented and supplemented sponges respectively, Fig. 4e,f). Uptake rates of DOC were driven by a 3-way interaction between the factors (F_(1,48)_ = 11.39, p = 0.001). Uptake rates of particulate organic carbon showed similar trends, yet without statistically robust support (POC, Tables S3 and S4).

**Figure 5:**
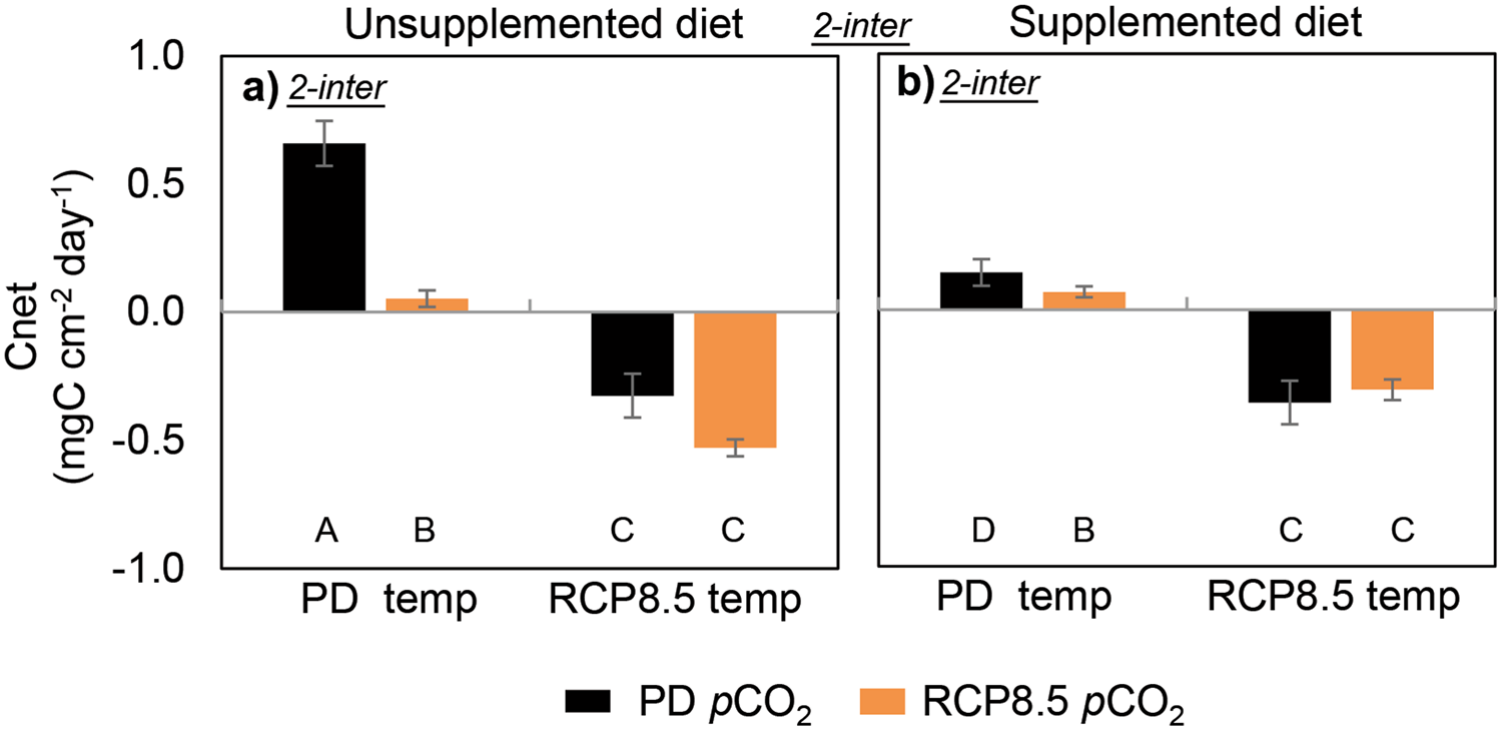
Daily net carbon surplus (C_net_) of * Cliona orientalis* at Heron Island on the southern Great Barrier Reef receiving an unsupplemented (**a**) and supplemented diet (**b**) under independent and concurrent simulation of warming and acidification from present-day (PD) levels to Representative Concentration Pathway 8.5 (RCP8.5) summer levels of the year 2100 (mean ± SEM). The carbon surplus incorporates autotrophic inputs (photosynthetically fixed carbon), heterotrophic inputs (net uptake of dissolved and particulate organic carbon) and respiratory output (carbon used for maintenance, growth etc.) over a 24 h period after 4 weeks of exposure to full treatment conditions. The response of C_net_ to the treatments was driven by 2-way interactive effects (*2-inter*) for each combination of the 3 factors. The capitalized letters beneath the bars indicate statistical differences.

At PD temperature and *p*CO_2,_ supplemented sponges had a lower daily net carbon surplus than unsupplemented sponges (C_net_, Fig. 5 and Table S4; F_(1,48)_ = 15.19, p < 0.001 and F_(1,48)_ = 20.13, p < 0.0001). Regardless of diet or *p*CO_2_, only the sponges exposed to warming were net consumers of metabolic carbon i.e. the metabolic demand of bleached sponges surpassed the carbon that could be autotrophically or heterotrophically harvested. Acidification led to reduced C_net_ surplus at PD temperature levels when the sponges were not supplemented (F_(1,48)_ = 28.78, p < 0.0001), reflecting the loss in DOC uptake despite the increase in photosynthetic activity which only contributes carbon during light hours.

### Biomass

Regardless of diet or *p*CO_2,_ the sponges were able to build or maintain more organic biomass under PD temperature than under RCP8.5 temperature (on average 41.74 and 36.69 mg_(biomass)_/g_(core)_ respectively, F_(1,48)_ = 13.16, p < 0.001, Tables S3 and S4). Non-sponge organics were found to be on average 1.94 ± 0.3% of total organics (no difference between treatments) and were therefore considered negligible in this study. The spicular mass of the sponge cores formed a large fraction of the sponge biomass (approx. 35% of total sponge biomass) and did not differ between treatments.

### Sponge mortality

After physiological responses had been measured at week 4 and during the second half of the experiment, the tissue of all bleached sponges increasingly retracted to a small surface area of the core, despite regular removal of epibionts. Bare parts of the core were colonized by a diverse algal community consisting mainly of crustose coralline algae (CCA), *Derbesia* sp., *Ulva* sp., pennate diatoms and free-living dinoflagellates (D Bender-Champ, pers. comm.). In approximately half of the specimens exposed to simulated warming, bleaching resulted in complete mortality (z = 3.65, p < 0.001) (Fig. 2d,e). The first cases of complete mortality co-occurred amongst PD *p*CO_2_ and RCP8.5 *p*CO_2_ treatments 6 weeks after the onset of full treatment, regardless of dietary supplementation. Complete mortality was always preceded by bleaching, occurring between 3–6 weeks post bleaching. Dead sponge cores were inhabited by fungi and cyanobacteria (*Spirulina* sp.) in addition to the algal community described above (D Bender-Champ, pers. comm.).

**Figure 6:**
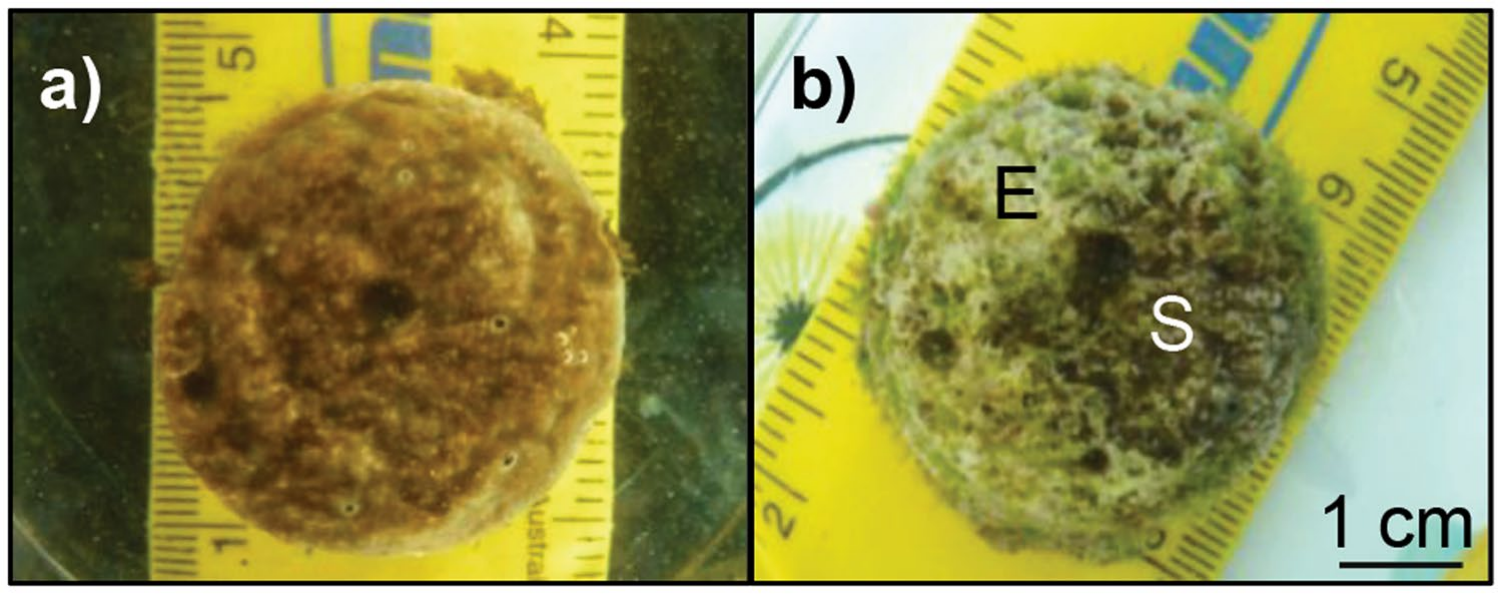
An example of partial mortality observed in a number of *Cliona orientalis* cores in the present-day temperature treatments that did not receive a supplemented diet. At the start of the experiment and at week 4 during the physiological measurements, sponge tissue covered the entire surface of the core (**a**). At the end of the experiment 10 weeks later, the tissue of several cores had retracted to a smaller surface area, exposing the CaCO_3_ substrate (**b**). This process may be a reversible result of stress as indicated by similar observations in other species^89^. Partial mortality was not observed in any of the sponges that received a supplemented diet. E = Exposed CaCO_3_ substrate, S = Sponge tissue.

Regardless of diet and *p*CO_2_, all specimens exposed to PD temperatures survived beyond the end of the summer in the experiment, without any signs of bleaching. However, in the unsupplemented treatment the substrate of some of these specimens had become partly exposed by the end of the experiment due to sponge tissue loss (partial mortality, Fig. 6). Here, partial mortality and consecutive algal settlement was found in 2 out of 5 of the PD *p*CO_2_ and 3 out of 5 of the RCP8.5 *p*CO_2_ sponges (z = 1.5806, p = 0.057 and z = 2.0698, p = 0.019, respectively) and was an independent effect of diet. Supplemented sponges retained their tissue, but unsupplemented sponges lost about one third of their tissue cover, resulting in 5.20 ± 0.78 cm^2^ live tissue compared to 8.23 ± 0.65 cm^2^ in supplemented sponges at the end of the experiment (two-way ANOVA, F_(1,16)_ = 7.96, p = 0.015). The surface tissue of supplemented sponges also appeared healthier and thicker.

## Discussion

Exposure of the photosymbiotic sponge *Cliona orientalis* to RCP8.5 summer projections for the year 2100 at Heron Island led to decreases in biomass and rates of bioerosion, autotrophy, heterotrophy and survival. These responses were driven by resource deprivation of both the host and the symbiont and were primarily caused by impacts of simulated warming, but also acidification, even when additional heterotrophic food sources were provided. Excavating sponges are often regarded as “winners” on disturbed coral reefs under projected future conditions^11^. However, based on the combined results of the current experiment, under “business-as-usual” CO_2_ emissions *C*. *orientalis* and possibly other similar photosymbiotic excavating sponges are expected to suffer losses at the end of the current century comparable to those projected for scleractinian corals^22^.

Extrapolating measured present-day summer rates of substrate removal by *C*. *orientalis* to an annual mean resulted in a total bioerosion rate of 12.4 kg CaCO_3_ m^−2^ year^−1^ when the diet was not supplemented, which is consistent with earlier results from similarly dense substrates^56^. Our study demonstrated a 35% stimulation of total sponge bioerosion by the availability of food, purportedly also allowing faster expansion as would be expected in nutrient-rich environments such as inshore reefs^35^. Our results do not imply a sustained increase in bioerosion performance by photosymbiotic sponges in future oceans however, mainly due to observed adverse impacts of simulated warming. We suggest that bioerosion rates were mainly reduced by the failure of the photosynthesis of the symbionts, highlighting the importance of the symbiosis to supply energy for bioerosion^38–40^. Previously documented temperature effects on bioerosion of *Cliona* species have shown variable responses^42, 45, 71, 72^. Our results are in accordance with the reduction of bioerosion rates under warming reported from a short-term experiment using the same species^45^. Simulated acidification co-occurred with heterotrophic carbon losses in our study, which may have resulted in the loss of a clear response through bioerosion as opposed to previous studies: Bioerosion by *C*. *orientalis* significantly increased under similar acidification in a 3-day closed system experiment (chemical bioerosion measured through alkalinity changes)^45^, in a 10-day flow-through experiment (total bioerosion measured through buoyant mass changes)^20^ and in a 8-week flow-through experiment over Austral spring (both of the above measures)^44^. We hypothesize that in our study over Austral summer, the longer-term losses to the sponge’s carbon budget under acidification may have lessened the energy invested into bioerosion, thereby decoupling acidification effects from erosion enhancement. We measured bioerosion through buoyant mass changes, but identifying chemical and mechanical rates in future studies could better elucidate potential acidification impacts.

Bleaching of *C*. *orientalis* tissues has previously been shown under concurrent simulation of RCP8.5 warming and acidification^44^. Here, we provide evidence that our observation of bleaching was caused by warming above the mean summer water temperature. Whether the bleaching is a result of oxidative stress and mechanisms similar to those established for the cnidarian-*Symbiodinium* partnership^73^ remains to be explored, but our results show that the bleached sponges experience reduced holobiont productivity and increased mortality, as is known for bleached corals^73^. The decrease of photosynthetic activity translates into reduced access to resources, yet even though bleached specimens had lower biomass and no symbiont population, they maintained a considerable respiratory demand (metabolic needs increase inherently with temperature rise), which would have represented an additional resource drain. To date, bleaching of *Cliona* spp. has been considered a rare event *in* or *ex situ* ^10^. In October 2015 a natural bleaching event was reported for the first time for a clionaid sponge in the lower Florida Keys^74^, but the sponges were only partially bleached and appeared to survive and recover (M Hill, pers. comm.). An unknown encrusting *Cliona* sp. exhibited impaired photosynthesis during a heating event in March 2013^75^ (MMM + 1 °C or more) in depths down to 15 m near Onslow, NW Australia (CHL Schönberg, pers.comm.). In our experiment, the sponges began to pale prior to the full establishment of the 2100 climate scenarios, indicating that at least partial or occasional bleaching could become more common in natural populations under similar warming events before the end of the current century.

Acidification stimulated higher photosynthetic rates in unbleached specimens that were not diet-supplemented, possibly because less resources had to be invested into the conversion of HCO_3_^−^ to CO_2_ for use by the Rubisco enzyme^76^. No such effect was found when the sponges received a supplemented diet since they may have been less dependent on autotrophic inputs, despite stable respiration that could otherwise provide an alternative source of CO_2_ to the symbiont^76^.

With regards to organic carbon uptake, an increase in heterotrophic feeding of the bleached sponges could have served to compensate for the loss of autotrophic carbon. However, sponges in the heated treatments had reduced rather than increased carbon uptake rates. Filter feeding is an energetically costly process^77^ that could be rendered unsustainable in the bleached sponges. Negative effects of temperature on sponge feeding have been reported for the Great Barrier Reef (GBR) sponge *Rhopaloeides odorabile*, with filtration efficiency, pumping rate and choanocyte chamber density and size reduced at 31 °C (MMM + 2 °C)^78^. POC uptake in our sponges was only insignificantly reduced, yet choanocyte functioning and water pumping may also facilitate DOC uptake^79^, which could explain the losses observed here under simulated warming. Acidification similarly reduced uptake of DOC, which may point towards a trade-off between autotrophy (due to stimulation of photosynthesis under elevated *p*CO_2_) and heterotrophy. Alternatively, prolonged high levels of H^+^ may have had deleterious effects on the filter-feeding capacity of the sponge, for example by affecting mitochondrial ATP recycling which is crucial to the flagellar beating^80^. Further investigations are necessary to confirm what caused the observed decrease in carbon uptake under acidification.

In treatments with either warming or acidification, dietary supplementation slightly ameliorated the effect of the other factors; the sponges took advantage of the elevated concentration of organics and more organic carbon was incorporated. Sponges in present-day conditions did not respond in the same way. Compared to supplemented sponges that were not in need of food, unsupplemented sponges may have been relatively starved (only particles up to 10 μm in diameter were retained in unsupplemented tanks) and were therefore filtering more actively (i.e. taking up more carbon from the seawater) when assessed over a 24h-period at the height of summer. The assumption of resource deficiency is also supported by the partial mortality found towards the end of the experiment in unbleached sponges that did not receive a supplemented diet.

All bleached sponges had lower organic biomass, which will in part explain the reduction in total bioerosion rates and respiration in the heated treatments. Bleached *C*. *orientalis* in previous experiments increased biomass^44, 46^, but this may have been due to a lag effect after an initial stimulation of growth earlier in spring when the symbionts were still present (JKH Fang, pers. comm.). The duration and the severity of our experimental warming went beyond physiological thresholds, and bleached sponges suffered biomass losses due to reduced autotrophy and heterotrophy. Supplementary feeding with protein-rich organics was expected to stimulate growth, since the extent to which the sponge holobiont can utilize photosynthetically transferred or heterotrophically attained carbon beyond its respiratory needs in the oligotrophic reef waters may depend on the availability of commonly limiting nutrients such as nitrogen^81^. No such effect was observed, but instead the resources gained from the extra nutrition may have been redirected towards bioerosion activity. Alternatively, the sponges may have not been nitrogen-limited in the first place, yet the partial mortality observed in the unsupplemented treatments is contraindicative.

Even though spiculogenesis is considered energy-demanding^82, 83^, the mass of siliceous spicule was not affected by any of the treatments, which confirms previous results^17, 44^. The formation of one demosponge spicule may take approximately one week and an even longer lag period needs to be considered when looking for environmental effects on spicules^84^.

A daily net carbon surplus was only realized under present-day temperatures, and that surplus was significantly reduced with acidification, since organic carbon uptake decreased. Overall, the physiological measurements revealed carbon deprivation in the bleached sponges, which explains their subsequent mortality. Heterotrophy by itself does not appear to be sufficient for the energetic needs of *C*. *orientalis* (also shown by ref. 46). We conclude that the symbiosis between the sponge and its dinoflagellates is neither facultative nor merely beneficial to bioerosion performance alone, but it is vital to the survival of the holobiont in several ways.

For marine sponges, interactive effects of environmental factors such as warming, acidification and food availability remain largely unknown and vary between species depending on the natural conditions that they are adapted to^45, 85^. The lack of and the need for related studies has recently been highlighted in order to quantify present trends of carbonate budgets and to provide modelled trajectories for different scenarios to facilitate management^11^. Our experiment assessed the effects of simulated warming, acidification and diet supplementation on the bioerosion efficiency and survival of *C*. *orientalis* separately and in combination. Overall, warming appears to be a more important factor in determining the physiological thresholds of the sponge, and increased bioerosion rates observed in previous short-term acidification-only experiments^11^ may not realistically reflect future developments under coexisting ocean warming and acidification. In non-bioeroding sponges from the GBR, the effects of RCP8.5 warming were also shown to be physiologically more important than acidification effects^86^, but Caribbean sponges exposed to similar warming and acidification for 24 days remained largely unaltered^85^. Our study suggests that future climate conditions may temporarily incur increased bioerosion rates at intermediate levels of environmental change, but that ultimately escalating environmental conditions under presently predicted “business-as-usual” fossil fuel usage will cause photosymbiotic clionaids to fail along with other benthic organisms. This supports a parabolic rather than a linear response of bioeroding sponges to future change^11^. Bioerosion and biomass maintenance are not the only energetically-costly activities of *C*. *orientalis*; other costly processes such as reproduction or competition for space^39^ could also be impacted by climate change, thereby possibly further reducing the likelihood of survival in this species.

Climate simulations on coral reef assemblages from the southern GBR have provided evidence that firstly, there has been little adjustment of corals to changes over the past 100 years, and secondly, 100 years from now corals are unlikely to calcify or survive over RCP8.5 summers (when temperatures and seawater *p*CO_2_ concentrations are at their seasonal highest) and beyond^47^. To date, excavating sponges were often considered more tolerant to future changes than scleractian corals^10^. However, our study stresses that there are limits especially to the temperature but also the *p*CO_2_ conditions that *C*. *orientalis* can tolerate, and that, despite subtle benefits from higher food availability, future summers can be expected to have adverse effects on the bioerosion capacity, general physiology and ultimately survival of the sponge. Keeping in mind the limitations of extrapolating from simulations to the field, we nevertheless cannot support that excavating sponges such as *C*. *orientalis* are still likely to be “winners” in 2100.

We acknowledge that the present results may only be relevant for excavating sponges that are symbiotic with *Symbiodinium* and that other excavating sponges might show different response patterns. This remains an essential area to explore if we are to gain a complete understanding of the implications of a warmer, more acidic and more eutrophic ocean for these key reef organisms. It is also important to note that our study did not identify the temperature threshold for the loss of *C*. *orientalis* from coral reefs like those of Heron Island and the southern Great Barrier Reef, or how well *C*. *orientalis* is performing as climate change intensifies in the interim, causing mass coral mortality events^87^ and thus increasing the availability of bioerosion substrate.

## Electronic supplementary material

Supplementary Information
