# Supplementary material for: Sponge bioerosion on changing reefs: ocean warming poses physiological constraints to the success of a photosymbiotic excavating sponge
Source: Sci Rep. 2017 Sep 6;7:10705. doi: 10.1038/s41598-017-10947-1 (PMC5587736; doi:10.1038/s41598-017-10947-1)
Supplement: Supplementary file 1 — Supplementary Information [file 41598_2017_10947_MOESM1_ESM.pdf]

Supplementary information

Sponge bioerosion on changing reefs: ocean warming poses physiological constraints to the success of a photosymbiotic excavating sponge

Michelle Achlatis*, Rene M. van der Zande, Christine H.L. Schönberg, James K.H. Fang, Ove Hoegh-Guldberg, Sophie Dove

*Corresponding author:
Michelle Achlatis, tel +61 7 336 59154, fax: +61 7 336 54755, email: m.achlatis@uq.edu.au

Table of contents

Supplementary Methods 2

Simulation system 2

Treatment conditions 2

Buoyant mass conversion 3

Metabolic oxygen and carbon flux 3

Density of the sponge cores 4

Supplementary Tables 5

Table S1. Seawater parameters at week 4 of the experiment 5

Table S2. Seawater chemistry parameters measured throughout the experiment 6

Table S3. Particulate organic carbon (POC) uptake and sponge biomass 10

Table S4. Results of 3-way factorial ANOVA 11

Supplementary Figures 16

Figure S1. Daily temperature profiles 16

Figure S2. Weekly pH profiles 17

Figure S3. Schematic illustration of a $CaCO_3$ sample eroded by an excavating sponge 18

Figure S4. An example of overlapping responses produced by the 6 genotypes 19

References 20

Supplementary Methods

Simulation system. The ocean warming and acidification simulation system established at the Heron Island Research Station on the southern GBR employed computer control systems to replicate diel and seasonal variations of temperature and *p*CO_2 conditions on the reef in an experimental setting[1,2]. Projected increases of temperature and *p*CO_2 were applied as offsets to a baseline produced by 2 or 3 hourly measurements at a reference site on Heron Island's reef crest (Harry's Bommie) over the summer of the previous year[3]. The offsets were established according to projected conditions for the year 2100 based on the greenhouse gas concentration trajectory RCP8.5[4,5], the endpoint of which is equivalent to the A1FI scenario[6].

The scenarios were continuously reproduced and monitored in four individual 8000 L sumps (residence time: 4-6 hours). The sumps were darkened, insulated with a rubber seal and inspected regularly. Seawater temperature in the sumps was controlled by heater-chillers (HWP017, Rheem, Rydalmere, Australia, equipped with Eurotherm 3216 temperature regulators, Invensys Process Systems, Clayton, Australia). The appropriate amounts of CO_2 gas (food grade), compressed air and "CO_2-free" air were injected into the sumps through an array of gas solenoid valves. Compressed air was stripped of CO_2 in desiccant columns containing soda lime (medical grade Spherasorb, Intersurgical, Berkshire, UK). A non-dispersive infrared (NDIR) sensor (CO_2-Pro, Pro-Oceanus, Nova Scotia, Canada, accuracy ±0.5% of CO_2 concentration, resolution 0.01ppm) measured the sumps in continuous rotation. The sensor was calibrated using Coregas (Yennora, Australia) span gases (102.3, 403.7, 613.8 and 1198 ppm CO_2). Every 24hours, an automatic zero compensation circuit removed all CO_2 from the detector prior to a zero-reference measurement, providing a stable long-term measurement baseline.

The timing and rate of heating, chilling and gas dosing in each sump were regulated by a central computer system according to the baseline from the reference site using custom-made software based on LabVIEW (National Instruments, Austin, USA). The software for temperature regulation was created by the School of Information Technology and Electrical Engineering (The University of Queensland) and the software for *p*CO_2 regulation by SciWare (Brisbane, Australia). The software was also capable of triggering an alarm system if offsets deviated from the given setpoints.

The treated water flowed continuously from the sumps (20L/min per sump) through individual pipes to 40 L experimental aquaria which contained the sponge replicates and were equipped with HOBO Pendant temperature loggers (Onset, Bourne, USA), Odyssey light loggers (Dataflow Systems, Christchurch, New Zealand) and small wave makers. pH was continuously monitored in one experimental tank per treatment using InPro4501VP X pH sensors (Mettler Toledo, Victoria, Australia) connected to a monitoring system (ACQ110, Aquatronica, Reggio Emilia, Italy). These sensors were rotated between tanks of the same treatment every 4th day. In addition, weekly pH (NBS scale) measurements were made in all 24 tanks at 11:00 and at 20:00 using a pHep sensor freshly calibrated (HI 98128, Hanna Instruments, Rhode Island, USA) (Fig. S2). Adjacent tanks with the same scenarios contained Pt100 temperature sensors (RS components, Wetherhill Park, Australia) that provided feed-back measurements to the sump controller. No feedback measurements were provided for pH/*p*CO_2, allowing a minimal extent of local *p*CO_2 regulation due to metabolic activity of the sponges in the experimental tanks.

The experimental elevation of temperature and *p*CO_2 are referred to as simulated warming and acidification respectively in the main text, while acknowledging the relatively shorter timescale of the experimental elevations compared to oceanographic changes[7].

Treatment conditions. During the experiment, sunrise time shifted from 05:10 to 05:50 h and sunset from 18:40 to 18:15 h, with in tank light intensities maximizing at mid-day (on average

463 ± 21 μmol quanta m^{-2} s^{-1}). After gradual adjustment of the treatments, full experimental conditions were reached on the 1st of January 2015, when daily mean water temperatures were 26.3 ± 0.3°C for the present-day (PD) temperature scenarios and 29.9 ± 0.1°C for the future (RCP8.5) temperature scenarios (mean ± SEM, Fig. S1). A snapshot of seawater chemistry parameters at week 4 (when most physiological measurements were performed) is given in Table S1, whereas Table S2 summarizes pH, total alkalinity and pCO_2 measurements made in the aquaria on other dates throughout the experiment. At the end of the experiment (9th of March 2015), which roughly coincided with the end of the Austral summer, temperature had decreased to approximately the same level as prevalent during week 1 (Fig. S1). Upstream sump pCO_2 at midday over the 10-week experimental period ranged from 428 ± 12 μatm to 519 ± 10 μatm for the PD pCO_2 scenarios and from 924 ± 5 μatm to 1053 ± 9 μatm for the RCP8.5 pCO_2 scenarios. Downstream midday pH in the experimental aquaria ranged from 7.98 to 8.27 for the PD pCO_2 scenarios and from 7.79 to 7.99 for the RCP8.5 pCO_2 scenarios.

Total alkalinity (A_T) of the experimental aquaria was measured with Gran titration using an automated titrator (T50, Mettler Toledo, Langacher, Switzerland) calibrated daily with pH NBS scale buffers (Radiometer analytical, Lyon, France) and Dickson's Standards (University of California, San Diego, USA)[8].

Buoyant mass conversion. To express total bioerosion rates of the sponge in mg $CaCO_3$ cm^{-2} core area day^{-1}, the measured changes in buoyant mass (BM) over time where converted to changes in $CaCO_3$ mass of the cores (M_{CaCO3}). This was made possible through a linear regression analysis between buoyant mass and $CaCO_3$ mass as measured from sponge and control cores at week 4 of the experiment, after having assessed the data for homoscedacity and the residuals for normality. Equation (1) expresses the regression found between the two variables (r^2=0.95, p<0.001, n=70):

$$\log(BM) = \log(M_{CaCO3}) - 0.196 \quad (1)$$

Metabolic oxygen and carbon flux. A theoretical curve of daily net oxygen production/ consumption was constructed based on the average daily irradiance profile during the experimental phase (see [9] for details). Net oxygen production by *C. orientalis* can saturate at around 300 μmol photons m^{-2} s^{-1} [(9)], which was attained between 09:00 and 14:00 h. Oxygen production was assumed to decrease linearly from max P_{net} to R_{dark} between 14:00 and 18:00 h, maintain R_{dark} levels during darkness (18:00 to 05:00 h) and linearly increase from R_{dark} to max P_{net} between 05:00 and 09:00 h. Oxygen flux during light, dark and transitioning hours was defined as time integrals and geometrically calculated from the relevant areas of the theoretical curve (see Fig. 1 in [9]). Oxygen flux was then summed over 24 h and converted to carbon flux (mg C cm^{-2} day^{-1}) based on the stoichiometric balance of photosynthesis (0.375 g carbon per g oxygen), applying a photosynthetic quotient of 1.1 and a respiratory quotient of 0.9[9,10].

To quantify the flux of particulate and dissolved organic carbon (POC and DOC respectively), sponges were incubated in confined seawater (5 L incubation chambers) over 24 hours. Seawater samples were collected in glass vials (pre-combusted and washed with 1N hydrochloric acid and Milli-Q water), which were sealed and immediately frozen for later analysis. Each of the defrosted seawater samples was filtered through a pre-combusted GF/F filter (pore size 0.7 μm) to separate particles >0.7 μm along with POC onto the filter while collecting the filtrate, which contains the DOC[11]. Subsequently, Milli-Q water was washed through the filters to remove salts, followed by 0.1N HCl to remove any inorganic carbon and Milli-Q again before drying the filters at 60°C overnight. Dried filters were weighted to ±0.001 mg and wrapped into large tin capsules. Quantitative combustion was performed at the School

of Chemistry and Molecular Biosciences at the University of Queensland with a FLASH 2000 CHNS/O Analyzer. Blank filters were used to correct the measured values. To quantify DOC, the filtrate was analysed at the Advanced Water Management Centre at the University of Queensland using a Total Organic Carbon Analyzer (TOC-L CSH with TNM-L TN unit, Shimadzu). The samples were first acidified using HCl and aerated to remove inorganics, and non-purgeable organic carbon was measured immediately thereafter[12].

Density of the sponge cores. The bulk density of the sponge cores was quantified at the beginning of the experiment and at week 4 of the experiment. Subsamples of $CaCO_3$ with sponge infestation were isolated and reconditioned overnight in distilled water on a shaker table. Without exposing the samples to air, they were transferred to 12% sodium hypochlorite for 4 h and rinsed and buoyant weighed in distilled water (buoyant mass in water = $m_{buoyant}$). Subsequently the samples were drip-dried, weighed in air (wet mass = m_{wet}) and dried at 60°C to a stable mass (dry mass = m_{dry}). Following Archimedes' principle, the volumes of the skeletal matrix of the cores ($V_{skeletal\ matrix}$, Fig. S3) were estimated [Equation (2), where d_{water} is the density of distilled water (1.00 g cm^{-3} at 22°C)[13]]. Since the samples were perforate (Fig. S3), pore volumes (V_{pores}) [Equation (3)] were added to the skeletal matrix volumes in order to calculate the bulk volumes of the cores (V_{bulk}) [Equation (4)]. Bulk densities (d_{bulk}, g cm^{-3}) and porosity (Por, %) were then calculated using Equation (5) and (6) respectively.

$$V_{skeletal\ matrix} = (m_{dry} - m_{buoyant}) / d_{water} \qquad (2)$$

$$V_{pores} = (m_{wet} - m_{dry}) / d_{water} \qquad (3)$$

$$V_{bulk} = V_{skeletal\ matrix} + V_{pores} \qquad (4)$$

$$d_{bulk} = m_{dry} / V_{bulk} \qquad (5)$$

$$Por = (100 \times V_{pores}) / V_{bulk} \qquad (6)$$

Supplementary Tables

Table S1. Seawater parameters at week 4 of the experiment for each of the scenarios, irrespective of diet. *P*CO_2 was measured in upstream sumps (*p*CO_2 upstream), whereas temperature, total alkalinity (A_T) and pH_{NBS} (NBS scale) are given as means ± SEM of values measured in all downstream aquaria (n=6 for each T and *p*CO_2 combination, irrespective of diet). Values represent instantaneous averages at 11:00 h and at 20:00 h (light intensity 414 and 0 μmol quanta m^{-2} s^{-1} respectively) on 31/01/2015. Downstream *p*CO_2, aragonite saturation state (Ω_{Arag}), bicarbonate (HCO_3^-) and carbonate (CO_3^{2-}) were estimated using the software CO2calc 1.2.0[14] and therefore lack error bars. Conditions are abbreviated PD for present-day levels and RCP8.5 for future levels of temperature and *p*CO_2. For seawater parameters measured on other dates throughout the experiment see Table S2. (SW = seawater).

	PD temperature		RCP8.5 temperature	
	PD *p*CO_2	RCP8.5 *p*CO_2	PD *p*CO_2	RCP8.5 *p*CO_2
11:00 h				
pCO_2 upstream (μatm)	491 ± 2.4	919 ± 1.7	435 ± 2.5	942 ± 2.3
Temperature (°C)	28.1 ± 0.1	27.8 ± 0.1	31.3 ± 0.1	31.1 ± 0.1
A_T (μmol $kgSW^{-1}$)	2275.3 ± 0.4	2274.1 ± 0.6	2273.6 ± 1.1	2272.9 ± 1.1
pH_{NBS}	8.11 ± 0.01	7.88 ± 0.01	8.16 ± 0.01	7.90 ± 0.02
pCO_2 downstream (μatm)	488	909	429	916
HCO_3^- (μmol $kgSW^{-1}$)	1784	1961	1699	1949
CO_3^{2-} (μmol $kgSW^{-1}$)	199	127	233	133
Ω_{Arag}	3.22	2.05	3.84	2.25
20:00 h				
pCO_2 upstream (μatm)	390 ± 2.3	915 ± 1.8	406 ± 2.2	887 ± 2.1
Temperature (°C)	28.3 ± 0.1	28.6 ± 0.0	31.2 ± 0.0	31.0 ± 0.1
A_T (μmol $kgSW^{-1}$)	2227.0 ± 1.4	2229.7 ± 1.4	2228.8 ± 1.1	2229.9 ± 1.5
pH_{NBS}	8.10± 0.02	7.87± 0.01	8.14± 0.02	7.86± 0.01
pCO_2 downstream (μatm)	493	920	446	960
HCO_3^- (μmol $kgSW^{-1}$)	1755.098	1923.322	1687.998	1910.934
CO_3^{2-} (μmol $kgSW^{-1}$)	191.47	124.664	219.475	129.925
Ω_{Arag}	3.09	2.02	3.60	2.13

Table S2. Seawater chemistry parameters measured throughout the experiment in the downstream aquaria. pH (NBS scale) and total alkalinity (A_T, in µmol $kgSW^{-1}$) were measured using the Gran titration method[8]. Downstream pCO_2 (in µatm) in the experimental aquaria (Aq # refers to a specific aquarium for each measurement) was estimated using the software CO2calc 1.2.0[14] and compared to upstream sump pCO_2 for each timepoint. Conditions are abbreviated PD for present-day levels and RCP8.5 for future levels of temperature and pCO_2. (SW = seawater).

	Treatment					pCO_2				
Aq #	T	pCO_2	Feeding	pH	A_T	downstream	upstream	Date	Day/ Night	Time
9	PD	PD	U	8.119	2317	482	452	7/01/2015	Day	16:00
18	PD	PD	U	8.124	2328	477		7/01/2015	Day	16:00
6	PD	PD	S	8.128	2317	470		7/01/2015	Day	16:00
13	PD	PD	S	8.140	2321	456		7/01/2015	Day	16:00
4	PD	PD	U	8.122	2248	466	459	8/01/2015	Day	16:00
9	PD	PD	U	8.105	2244	488		8/01/2015	Day	16:00
18	PD	PD	U	8.120	2247	468		8/01/2015	Day	16:00
6	PD	PD	S	8.122	2245	465		8/01/2015	Day	16:00
13	PD	PD	S	8.120	2247	468		8/01/2015	Day	16:00
23	PD	PD	S	8.121	2245	466		8/01/2015	Day	16:00
9	PD	PD	U	8.097	2280	508	451	12/01/2015	Day	13:00
18	PD	PD	U	8.095	2280	511		12/01/2015	Day	13:00
6	PD	PD	S	8.112	2287	489		12/01/2015	Day	13:00
13	PD	PD	S	8.104	2281	498		12/01/2015	Day	13:00
4	PD	PD	U	8.212	2288	369	402	13/01/2015	Day	13:00
9	PD	PD	U	8.222	2287	358		13/01/2015	Day	13:00
18	PD	PD	U	8.241	2311	342		13/01/2015	Day	13:00
6	PD	PD	S	8.222	2292	359		13/01/2015	Day	13:00
13	PD	PD	S	8.273	2297	309		13/01/2015	Day	13:00
23	PD	PD	S	8.279	2298	304		13/01/2015	Day	13:00
4	PD	PD	U	8.098	2313	511	461	15/01/2015	Day	12:00
9	PD	PD	U	8.082	2310	533		15/01/2015	Day	12:00
18	PD	PD	U	8.099	2313	509		15/01/2015	Day	12:00
6	PD	PD	S	8.082	2311	533		15/01/2015	Day	12:00
13	PD	PD	S	8.078	2310	539		15/01/2015	Day	12:00
23	PD	PD	S	8.101	2311	506		15/01/2015	Day	12:00
4	PD	PD	U	8.081	2293	531	389	16/01/2015	Day	16:00
9	PD	PD	U	8.095	2290	510		16/01/2015	Day	16:00
18	PD	PD	U	8.129	2310	468		16/01/2015	Day	16:00
6	PD	PD	S	8.138	2295	454		16/01/2015	Day	16:00
13	PD	PD	S	8.160	2296	426		16/01/2015	Day	16:00
23	PD	PD	S	8.130	2290	463		16/01/2015	Day	16:00
4	PD	PD	U	8.109	2237	480	527	12/02/2015	Day	15:00
9	PD	PD	U	8.122	2235	463		12/02/2015	Day	15:00

18	PD	PD	U	8.114	2240	474		12/02/2015	Day	15:00
4	PD	PD	U	8.144	2280	441	404	16/02/2015	Day	16:00
9	PD	PD	U	8.148	2280	436		16/02/2015	Day	16:00
18	PD	PD	U	8.151	2283	433		16/02/2015	Day	16:00
4	PD	PD	U	8.166	2263	412	380	16/02/2015	Night	21:00
9	PD	PD	U	8.150	2260	430		16/02/2015	Night	21:00
18	PD	PD	U	8.164	2262	414		16/02/2015	Night	21:00
4	PD	PD	U	8.121	2262	466	487	5/03/2015	Day	14:00
9	PD	PD	U	8.119	2271	471		5/03/2015	Day	14:00
18	PD	PD	U	8.113	2266	478		5/03/2015	Day	14:00
6	PD	PD	S	8.129	2212	446		5/03/2015	Day	14:00
13	PD	PD	S	8.169	2212	398		5/03/2015	Day	14:00
23	PD	PD	S	8.159	2212	410		5/03/2015	Day	14:00
4	PD	PD	U	8.122	2275	468	518	8/03/2015	Day	12:00
9	PD	PD	U	8.119	2291	476		8/03/2015	Day	12:00
18	PD	PD	U	8.113	2294	484		8/03/2015	Day	12:00
6	PD	PD	S	8.175	2296	407		8/03/2015	Day	12:00
23	PD	PD	S	8.143	2296	446		8/03/2015	Day	12:00
2	PD	RCP8.5	U	7.942	2281	763	947	12/01/2015	Day	13:00
14	PD	RCP8.5	U	7.922	2279	804		12/01/2015	Day	13:00
7	PD	RCP8.5	S	7.879	2276	898		12/01/2015	Day	13:00
21	PD	RCP8.5	S	7.921	2277	805		12/01/2015	Day	13:00
2	PD	RCP8.5	U	7.918	2232	802	881	24/01/2015	Night	18:00
12	PD	RCP8.5	U	7.911	2234	818		24/01/2015	Night	18:00
14	PD	RCP8.5	U	7.909	2238	824		24/01/2015	Night	18:00
7	PD	RCP8.5	S	7.909	2240	824		24/01/2015	Night	18:00
17	PD	RCP8.5	S	7.912	2243	819		24/01/2015	Night	18:00
21	PD	RCP8.5	S	7.910	2241	823		24/01/2015	Night	18:00
2	PD	RCP8.5	U	7.860	2307	969	934	26/01/2015	Day	13:00
12	PD	RCP8.5	U	7.916	2293	832		26/01/2015	Day	13:00
14	PD	RCP8.5	U	7.912	2294	841		26/01/2015	Day	13:00
7	PD	RCP8.5	S	7.871	2304	941		26/01/2015	Day	13:00
17	PD	RCP8.5	S	7.919	2289	824		26/01/2015	Day	13:00
21	PD	RCP8.5	S	7.913	2288	836		26/01/2015	Day	13:00
2	PD	RCP8.5	U	7.898	2267	873	922	13/02/2015	Day	11:00
12	PD	RCP8.5	U	7.916	2270	834		13/02/2015	Day	11:00
14	PD	RCP8.5	U	7.920	2273	826		13/02/2015	Day	11:00
2	PD	RCP8.5	U	7.845	2244	976	932	13/02/2015	Night	21:00
12	PD	RCP8.5	U	7.859	2241	939		13/02/2015	Night	21:00
14	PD	RCP8.5	U	7.875	2252	906		13/02/2015	Night	21:00
2	PD	RCP8.5	U	7.875	2256	925	912	14/02/2015	Day	15:00
12	PD	RCP8.5	U	7.904	2261	858		14/02/2015	Day	15:00

14	PD	RCP8.5	U	7.894	2258	880		14/02/2015	Day	15:00
2	PD	RCP8.5	U	7.887	2278	884	1008	23/02/2015	Night	21:00
12	PD	RCP8.5	U	7.879	2284	905		23/02/2015	Night	21:00
14	PD	RCP8.5	U	7.891	2284	878		23/02/2015	Night	21:00
7	PD	RCP8.5	S	7.871	2342	946	1006	3/03/2015	Day	10:00
17	PD	RCP8.5	S	7.823	2342	1071		3/03/2015	Day	10:00
21	PD	RCP8.5	S	7.848	2346	1006		3/03/2015	Day	10:00
7	PD	RCP8.5	S	7.861	2283	950	945	3/03/2015	Night	22:00
17	PD	RCP8.5	S	7.885	2275	889		3/03/2015	Night	22:00
21	PD	RCP8.5	S	7.865	2274	936		3/03/2015	Night	22:00
2	PD	RCP8.5	U	7.869	2249	904	1047	4/03/2015	Day	12:00
12	PD	RCP8.5	U	7.860	2262	931		4/03/2015	Day	12:00
14	PD	RCP8.5	U	7.866	2250	911		4/03/2015	Day	12:00
7	PD	RCP8.5	S	7.877	2222	875		4/03/2015	Day	12:00
17	PD	RCP8.5	S	7.862	2265	928		4/03/2015	Day	12:00
8	RCP8.5	PD	U	8.237	2338	352	398	15/01/2015	Day	14:00
15	RCP8.5	PD	U	8.201	2342	392		15/01/2015	Day	14:00
24	RCP8.5	PD	U	8.255	2346	335		15/01/2015	Day	14:00
3	RCP8.5	PD	S	8.189	2295	398		15/01/2015	Day	14:00
11	RCP8.5	PD	S	8.192	2295	394		15/01/2015	Day	14:00
20	RCP8.5	PD	S	8.164	2293	427		15/01/2015	Day	14:00
8	RCP8.5	PD	U	8.124	2312	482	427	17/01/2015	Day	11:00
15	RCP8.5	PD	U	8.147	2295	447		17/01/2015	Day	11:00
24	RCP8.5	PD	U	8.132	2316	472		17/01/2015	Day	11:00
3	RCP8.5	PD	S	8.155	2316	442		17/01/2015	Day	11:00
11	RCP8.5	PD	S	8.183	2319	408		17/01/2015	Day	11:00
20	RCP8.5	PD	S	8.156	2327	442		17/01/2015	Day	11:00
8	RCP8.5	PD	U	8.144	2258	445	463	5/02/2015	Day	15:00
15	RCP8.5	PD	U	8.148	2255	439		5/02/2015	Day	15:00
24	RCP8.5	PD	U	8.140	2255	449		5/02/2015	Day	15:00
8	RCP8.5	PD	U	8.129	2301	473	450	7/02/2015	Day	17:00
15	RCP8.5	PD	U	8.131	2301	471		7/02/2015	Day	17:00
24	RCP8.5	PD	U	8.142	2301	456		7/02/2015	Day	17:00
8	RCP8.5	PD	U	8.132	2246	458	459	9/02/2015	Night	21:00
15	RCP8.5	PD	U	8.146	2247	440		9/02/2015	Night	21:00
24	RCP8.5	PD	U	8.138	2245	450		9/02/2015	Night	21:00
8	RCP8.5	PD	U	8.150	2262	434	479	11/02/2015	Night	22:00
15	RCP8.5	PD	U	8.217	2259	357		11/02/2015	Night	22:00
24	RCP8.5	PD	U	8.200	2261	376		11/02/2015	Night	22:00
3	RCP8.5	PD	S	8.110	2300	498	430	2/03/2015	Day	16:00
11	RCP8.5	PD	S	8.142	2306	455		2/03/2015	Day	16:00
20	RCP8.5	PD	S	8.139	2306	460		2/03/2015	Day	16:00

3	RCP8.5	PD	S	8.158	2227	419	373	2/03/2015	Night	23:00
11	RCP8.5	PD	S	8.185	2225	387		2/03/2015	Night	23:00
20	RCP8.5	PD	S	8.141	2229	440		2/03/2015	Night	23:00
5	RCP8.5	RCP8.5	U	7.991	2316	694	954	7/01/2015	Day	16:00
16	RCP8.5	RCP8.5	U	7.982	2319	712		7/01/2015	Day	16:00
5	RCP8.5	RCP8.5	U	7.913	2292	857	950	20/01/2015	Day	13:00
16	RCP8.5	RCP8.5	U	7.910	2291	864		20/01/2015	Day	13:00
22	RCP8.5	RCP8.5	U	7.915	2290	852		20/01/2015	Day	13:00
1	RCP8.5	RCP8.5	S	7.923	2290	834		20/01/2015	Day	13:00
10	RCP8.5	RCP8.5	S	7.910	2289	863		20/01/2015	Day	13:00
19	RCP8.5	RCP8.5	S	7.917	2286	855		20/01/2015	Day	13:00
5	RCP8.5	RCP8.5	U	7.877	2281	933	938	22/01/2015	Day	17:00
16	RCP8.5	RCP8.5	U	7.879	2284	929		22/01/2015	Day	17:00
22	RCP8.5	RCP8.5	U	7.939	2282	791		22/01/2015	Day	17:00
1	RCP8.5	RCP8.5	S	7.921	2280	830		22/01/2015	Day	17:00
10	RCP8.5	RCP8.5	S	7.899	2282	880		22/01/2015	Day	17:00
19	RCP8.5	RCP8.5	S	7.895	2285	891		22/01/2015	Day	17:00
5	RCP8.5	RCP8.5	U	7.910	2258	851	958	5/02/2015	Day	15:00
16	RCP8.5	RCP8.5	U	7.899	2262	878		5/02/2015	Day	15:00
22	RCP8.5	RCP8.5	U	7.898	2259	879		5/02/2015	Day	15:00
5	RCP8.5	RCP8.5	U	7.893	2297	907	956	6/02/2015	Day	15:00
16	RCP8.5	RCP8.5	U	7.908	2297	872		6/02/2015	Day	15:00
22	RCP8.5	RCP8.5	U	7.890	2296	914		6/02/2015	Day	15:00
5	RCP8.5	RCP8.5	U	7.918	2245	824	944	8/02/2015	Night	22:00
16	RCP8.5	RCP8.5	U	7.925	2245	808		8/02/2015	Night	22:00
22	RCP8.5	RCP8.5	U	7.915	2244	830		8/02/2015	Night	22:00
1	RCP8.5	RCP8.5	S	7.921	2255	822	961	10/02/2015	Night	21:00
10	RCP8.5	RCP8.5	S	7.913	2259	842		10/02/2015	Night	21:00
19	RCP8.5	RCP8.5	S	7.915	2260	837		10/02/2015	Night	21:00
1	RCP8.5	RCP8.5	S	7.840	2342	1054	1113	21/02/2015	Day	11:00
10	RCP8.5	RCP8.5	S	7.818	2341	1116		21/02/2015	Day	11:00
19	RCP8.5	RCP8.5	S	7.873	2341	966		21/02/2015	Day	11:00

Table S3. Particulate organic carbon (POC) uptake and sponge biomass of *Cliona orientalis* at Heron Island on the southern Great Barrier Reef. Total sponge bioerosion, maximum net photosynthesis, dark respiration and dissolved organic carbon uptake are displayed in Fig. 3 and 4 of the main article. Outputs of statistical analyses of all variables are displayed in Table S4. Data were generated after 4 weeks of exposure to full treatment conditions of present day and RCP8.5 temperature and pCO_2, while receiving an unsupplemented or supplemented diet (mean ± SEM, n=7). The levels of temperature and pCO_2 are abbreviated PD for present-day levels and RCP8.5 for levels predicted for 2100.

		Unsupplemented diet		Supplemented diet	
		PD Temperature	RCP8.5 Temperature	PD Temperature	RCP8.5 Temperature
POC uptake (mg C cm^{-2} day^{-1})					
	PD pCO_2	0.25 ± 0.19	0.04 ± 0.03	0.05 ± 0.03	-0.03 ± 0.02
	RCP8.5 pCO_2	0.11 ± 0.07	-0.03 ± 0.02	0.11 ± 0.02	-0.02 ± 0.01
Sponge organic biomass ($mg_{(biomass)}/g_{(core)}$)					
	PD pCO_2	40.95 ± 1.76	38.28 ± 2.29	42.36 ± 2.12	36.66 ± 1.10
	RCP8.5 pCO_2	39.27 ± 1.32	36.25 ± 1.86	44.37 ± 2.72	35.60 ± 1.99
Spicular mass ($mg_{(biomass)}/g_{(core)}$)					
	PD pCO_2	22.84 ± 2.45	26.69 ± 2.69	23.16 ± 2.04	22.07 ± 3.69
	RCP8.5 pCO_2	24.96 ± 1.84	24.45 ± 1.30	23.47 ± 1.71	18.17 ± 2.89

Table S4. Results of 3-way factorial ANOVA and follow up tests where applicable for the key parameters presented in Figures 3-5 of the main article and Table S3. Temperature (T), pCO_2 and diet were the independent factors of the analysis. Simple main effects are reported following significant interactions of the factors, whereas main effects are reported when there is no interaction of the factors at the given level of comparison. The absolute width of the 95% confidence interval (CI) of the effect size of the single factors is shown for each variable. Temperature and pCO_2 are abbreviated PD for present-day levels and RCP8.5 for levels predicted for 2100. Max P_{net} = maximum net photosynthesis, R_{dark} = dark respiration, DOC = dissolved organic carbon, POC = particulate organic carbon, C_{net} = daily net carbon surplus, S = Supplemented diet, U = Unsupplemented diet. Data transformations: [a]log, [b]sqrt(x+3). *Indicates datasets with non-homogeneous variances where the significance level was reduced to 0.01 to minimize the risk of a Type I error[15]. All other data were evaluated at a 0.05 level of significance.

		ANOVA	Source of variation	DF	MS	F	P	Conclusions	Width of CI of effect size
**Sponge bioerosion rates (mg $CaCO_3$ cm^{-2} d^{-1})**									
1	Total[a]	3-way	T	1	0.115	4.54	0.038	PD>RCP8.5	0.023
			pCO_2	1	0.002	0.08	0.78		0.019
			Diet	1	0.205	8.07	0.007	S>U	0.006
			T × pCO_2	1	0.011	0.42	0.52		
			T × Diet	1	0.006	0.24	0.63		
			pCO_2 × Diet	1	0.000	0.00	0.98		
			T × pCO_2 × Diet	1	0.003	0.11	0.75		
			Error	48	0.025				
**Weight loss of control cores (mg $CaCO_3$ cm^{-2} d^{-1})**									
4	Total	3-way	T	1	0.120	0.93	0.34		0.022
			pCO_2	1	0.008	0.06	0.80		0.117
			Diet	1	0.004	0.03	0.86		0.083
			T × pCO_2	1	0.029	0.23	0.64		
			T × Diet	1	0.000	0.00	0.96		
			pCO_2 × Diet	1	0.130	1.00	0.32		
			T × pCO_2 × Diet	1	0.000	0.00	0.99		
			Error	48	0.129				

Sponge biomass ($mg_{(biomass)}/g_{(core)}$)

5	Organic mass	3-way	T	1	335.004	13.16	<0.001	PD>RCP8.5	0.241
			$p\mathrm{CO_2}$	1	6.261	0.25	0.62		0.698
			Diet	1	14.771	0.58	0.45		1.029
			T × $p\mathrm{CO_2}$	1	9.583	0.38	0.54		
			T × Diet	1	63.383	2.49	0.12		
			$p\mathrm{CO_2}$ × Diet	1	17.913	0.70	0.41		
			T × $p\mathrm{CO_2}$ × Diet	1	6.106	0.24	0.63		
			Error	48	25.456				
6	Spicular mass	3-way	T	1	8.153	0.20	0.66		1.953
			$p\mathrm{CO_2}$	1	12.087	0.29	0.59		1.111
			Diet	1	127.751	3.09	0.08		1.199
			T × $p\mathrm{CO_2}$	1	64.148	1.55	0.22		
			T × Diet	1	82.977	2.00	0.16		
			$p\mathrm{CO_2}$ × Diet	1	10.487	0.25	0.62		
			T × $p\mathrm{CO_2}$ × Diet	1	0.018	0.00	0.98		
			Error	48	41.394				

Oxygen flux (μM O_2 cm^{-2} h^{-1})

7	P_{net}[b]	3-way	T	1	10.754	189.11	<0.001		0.055
			$p\mathrm{CO_2}$	1	0.051	0.89	0.35		0.007
			Diet	1	0.069	1.22	0.27		0.020
			T × $p\mathrm{CO_2}$	1	0.136	2.39	0.13		
			T × Diet	1	0.065	1.14	0.29		

			pCO_2 × Diet	1	0.000	0.00	0.99		
			T × pCO_2 × Diet	1	0.369	6.48	0.014		
			Error	48	0.057				
7.1	Unsupplemented	Simple 2-way	T × pCO_2	1	0.467	8.21	0.006		
			Error	48	0.057				
7.1.1	PD pCO_2	Simple main effect	T	1	1.669	29.36	<0.0001	PD>RCP8.5	
			Error	48	0.057				
7.1.2	RCP8.5 pCO_2	Simple main effect	T	1	4.791	84.25	<0.0001	PD>RCP8.5	
			Error	48	0.057				
7.2	Unsupplemented	Main effect	pCO_2	1	0.339	5.96	0.018	PD<RCP8.5	
			Error	48	0.057				
7.3	Supplemented	Simple 2-way	T × pCO_2	1	0.029	0.51	0.48		
			Error	48	0.057				
7.3.1	PD pCO_2	Main effect	T	1	2.718	47.80	<0.0001	PD>RCP8.5	
			Error	48	0.057				
7.3.2	RCP8.5 pCO_2	Main effect	T	1	1.982	34.85	<0.0001	PD>RCP8.5	
			Error	48	0.057				
8	R_{dark}*	3-way	T	1	14.229	36.44	<0.0001	PD>RCP8.5	0.252
			pCO_2	1	0.354	0.91	0.35		0.027
			Diet	1	0.104	0.27	0.61		0.115
			T × pCO_2	1	0.243	0.62	0.43		
			T × Diet	1	0.014	0.04	0.85		
			pCO_2 × Diet	1	1.145	2.93	0.09		
			T × pCO_2 × Diet	1	0.032	0.08	0.78		
			Error	48	0.391				
**Carbon flux (mg C cm^{-2} d^{-1})**									
9	DOC uptake*	3-way	T	1	0.883	93.49	<0.001		0.075
			pCO_2	1	0.453	48.02	<0.001		0.135

			Diet	1	0.005	0.54	0.46		0.120
			T × pCO_2	1	0.365	38.66	<0.001		
			T × Diet	1	0.196	20.71	<0.001		
			pCO_2 × Diet	1	0.086	9.09	0.004		
			T × pCO_2 × Diet	1	0.108	11.39	0.001		
			Error	48	0.009				
9.1	Unsupplemented	Simple 2-way	T × pCO_2	1	0.434	46.01	<0.001		
			Error	48	0.009				
9.1.1	PD pCO_2	Simple main effect	T	1	1.338	141.76	<0.001	PD>RCP8.5	
			Error	48	0.009				
9.1.2	RCP8.5 pCO_2	Simple main effect	T	1	0.051	5.35	0.02		
			Error	48	0.009				
9.2	Supplemented	Simple 2-way	T × pCO_2	1	0.038	4.04	0.05		
			Error	48	0.009				
9.2.1	PD and RCP8.5 pCO_2	Main effect	T	1	0.124	13.10	0.001	PD>RCP8.5	
			Error	48	0.009				
9.3	PD T and pCO_2	Main effect	Diet	1	0.246	26.06	<0.001	S<U	
			Error	48	0.009				
9.4.1	PD T and U	Simple main effect	pCO_2	1	0.901	95.43	<0.001	PD>RCP8.5	
			Error	48	0.009				
9.4.2	PD T and S	Simple main effect	pCO_2	1	0.108	11.42	0.001	PD>RCP8.5	
			Error	48	0.009				
10	POC uptake*	3-way	T	1	0.021	5.62	0.02		0.166
			pCO_2	1	0.001	0.25	0.62		0.183
			Diet	1	0.001	0.38	0.54		0.203
			T × pCO_2	1	0.009	2.48	0.12		
			T × Diet	1	0.000	0.11	0.74		
			pCO_2 × Diet	1	0.000	0.12	0.73		
			T × pCO_2 × Diet	1	0.003	0.83	0.37		
			Error	48	0.004				

11	C_{net}	3-way	T	1	5.049	193.41	<0.0001		0.004
			pCO_2	1	0.585	22.43	<0.0001		0.011
			Diet	1	0.082	3.14	0.08		0.022
			T × *p*CO_2	1	0.235	9.01	0.004		
			T × Diet	1	0.387	14.81	<0.001		
			pCO_2 × Diet	1	0.516	19.76	<0.0001		
			T × *p*CO_2 × Diet	1	0.065	2.48	0.12		
			Error	48	0.026				
11.1	PD *p*CO_2	Simple main effect	T	1	3.887	148.91	<0.0001	PD>RCP8.5	
			Error	48	0.026				
11.2	RCP8.5 *p*CO_2	Simple main effect	T	1	1.493	57.18	<0.0001	PD>RCP8.5	
			Error	48	0.026				
11.3	PD temp	Simple main effect	*p*CO_2	1	0.751	28.78	<0.0001	PD>RCP8.5	
			Error	48	0.026				
11.4	RCP8.5 Temp	Simple main effect	*p*CO_2	1	0.041	1.57	0.21		
			Error	48	0.026				
11.5	PD temp	Simple main effect	Diet	1	0.396	15.19	<0.001	U>S	
			Error	48	0.026				
11.6	PD *p*CO_2	Simple main effect	Diet	1	0.526	20.13	<0.0001	U>S	
			Error	48	0.026				

Supplementary Figures

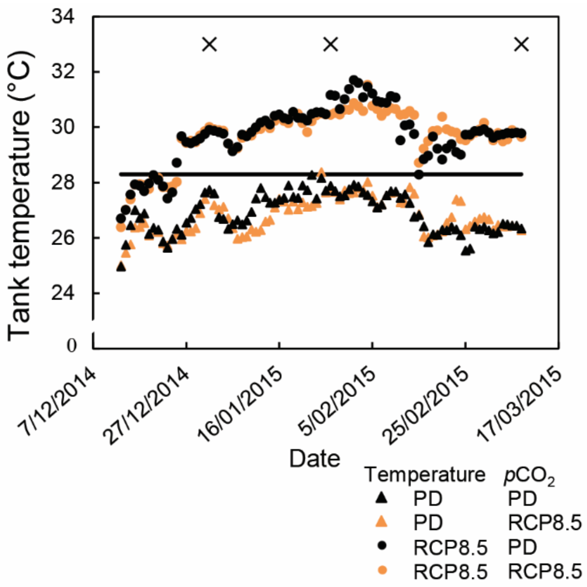

Figure S1. Daily temperature profiles obtained throughout the course of the experiment, pooled together for treatments supplemented with additional particulate food and unsupplemented treatments. Mean pCO_2 levels across the experiment were 493 ± 20 µatm (± SEM) for treatments with present day (PD) levels and 982 ± 17 µatm for treatments with Representative Concentration Pathway 8.5 (RCP8.5) levels. The solid line represents 1°C above the maximum monthly mean (MMM+1) established for the Heron Island region based on 50-km pixel satellite data[16]. Crosses represent the start of full treatment, the 4th week thereafter (when the physiological measurements described in the main text were performed) and the end of the experiment respectively.

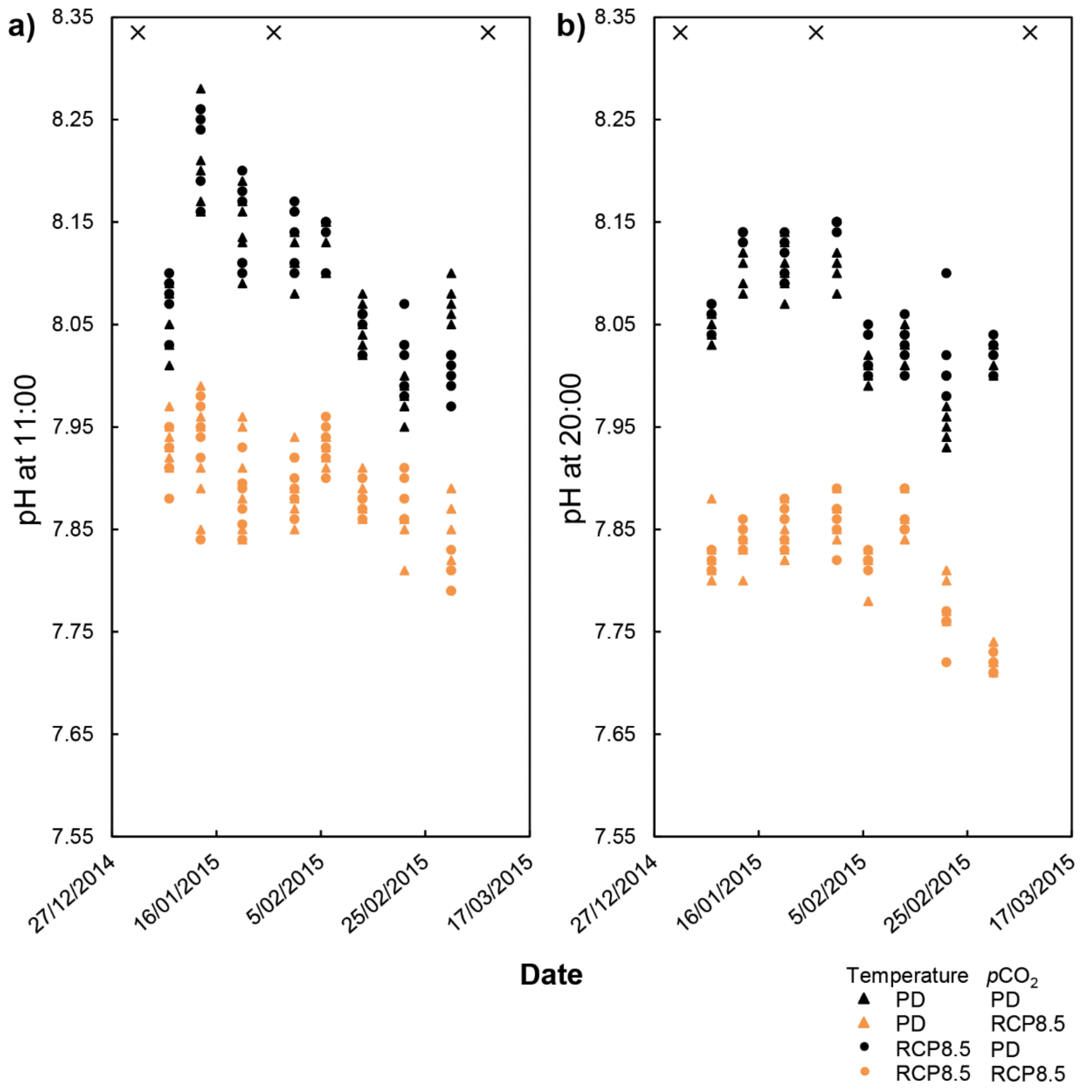

Figure S2. Weekly pH profiles (NBS scale) obtained throughout the course of the experiment, pooled together for treatments supplemented with additional particulate food and unsupplemented treatments. Measurements were made at **(a)** 11:00 h and **(b)** 20:00 h in each experimental aquarium. Crosses represent the start of full treatment, the 4th week thereafter (when the physiological measurements described in the main text were performed) and the end of the experiment respectively. The levels of temperature and pCO_2 are abbreviated PD for present-day levels and RCP8.5 for levels predicted for 2100.

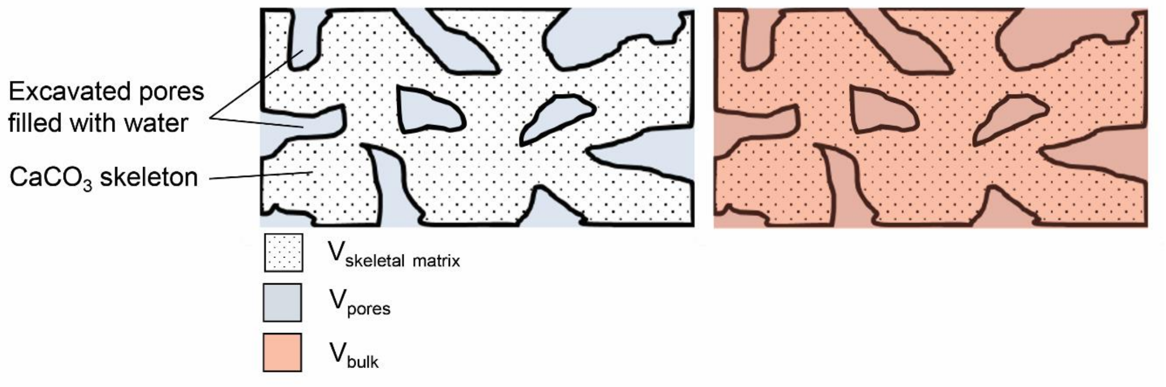

Figure S3. Schematic illustration of a $CaCO_3$ sample eroded by an excavating sponge. When submerged, pores created by excavation will be filled with water (in blue) after the sponge tissue has been removed. According to Archimedes' principle, a submerged object will appear to weigh less (buoyant mass in water = $m_{buoyant}$) than it would when dry in air (dry mass =m_{dry}) by an amount equal to the mass of the liquid it displaces (mass of displaced water= $V_{displaced\ water} \times d_{water}$, where $V_{displaced\ water}$ and d_{water} are the volume and density of the displaced water respectively). Buoyant weighing an object thus allows calculation of the volume of water that the object displaces, if the density of the water is known:

$$V_{displaced\ water} = (m_{dry} - m_{buoyant}) / d_{water}$$

For water-tight objects, the volume of the displaced water equals the volume of the object. Because the sponge is perforate, the volume of the displaced water equals the volume of the skeletal matrix of the sponge core only ($V_{skeletal\ matrix}$). Thus the pore volume must be added to the volume of the skeletal matrix before the bulk volume (V_{bulk}), and by this bulk density of the $CaCO_3$ eroded by the sponge can be measured.

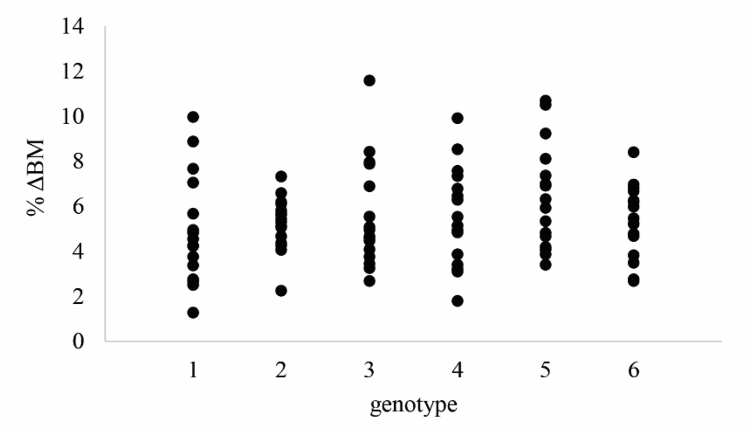

Figure S4. An example of overlapping responses produced by the 6 genotypes used in the experiment. For all variables, no single genotype or group of genotypes deviated from the others in response to the treatments in a way that would substantially bias the analysis. This example illustrates the percentage change in buoyant mass (%ΔBM) of the 6 different genotypes over the first 4 weeks of the experiment.

References

1. Dove, S. G. *et al.* Future reef decalcification under a business-as-usual CO_2 emission scenario. *Proc. Natl. Acad. Sci. USA.* **110,** 15342–15347 (2013).
2. Fang, J. K. H. Combined effects of ocean warming and acidification on the marine excavating sponge *Cliona orientalis* Thiele, 1900. PhD thesis, The Univeristy of Queensland (2013).
3. Commonwealth Scientific and Industrial Research Organisation and Pacific Marine Environmental Laboratory Carbon Program. Available at: https://www.pmel.noaa.gov/co2/story/Heron+Island (date of access Nov 2016).
4. IPCC. *Climate Change 2013: The Physical Science Basis. Contribution of Working Group I to the Fifth Assessment Report of the Intergovernmental Panel on Climate Change. (eds Stocker T.F. et al.), (Camb. Univ. Press, 2013).*
5. IPCC. *Climate Change 2014: Impacts, Adaptation, and Vulnerability. Contribution of Working Group II to the Fifth Assessment Report of the Intergovernmental Panel on Climate Change. (eds Field C.B. et al.),* (*Camb. Univ. Press,* 2014).
6. IPCC. *Special Report on Emissions Scenarios (eds Nakicenovic N., Swart R.),* (*Camb. Univ. Press,* 2000).
7. McElhany, P. CO_2 sensitivity experiments are not sufficient to show an effect of ocean acidification. *ICES J. Mar. Sci.,* doi:10.1093/icesjms/fsw085 (2016).
8. Kline, D. I. *et al.* A short-term in situ CO_2 enrichment experiment on Heron Island (GBR). *Sci. Rep.* **2,** 1–9 (2012).
9. Fang, J. K. H. *et al.* Effects of ocean warming and acidification on the energy budget of an excavating sponge. *Glob. Chang. Biol.* **20,** 1043–1054 (2014).
10. Muscatine, L., McCloskey, L.R. & Marian, R.E. Estimating the daily contribution of carbon from zooxanthellae to coral animal respiration. *Limnol. Oceanogr.* **26,** 601–611 (1981).
11. Yahel, G., Sharp, J. H., Marie, D., Hase, C. & Genin, A. In situ feeding and element removal in the symbiont-bearing sponge *Theonella swinhoei*: Bulk DOC is the major source for carbon. *Limnol. Oceanogr.* **48,** 141–149 (2003).
12. Findlay, S. *et al.* Total carbon analysis may overestimate organic carbon content of fresh waters in the presence of high dissolved inorganic carbon. *Limnol. Oceanogr. Methods* **8,** 196–201 (2010).
13. Lide, D.R. *CRC Handbook of Chemistry and Physics*. (CRC Press, 2006).
14. Robbins, L.L., Hansen, M.E., Kleypas, J.A., Meylan S.C. CO_2calc - A user-friendly seawater carbon calculator for Windows, Max OS X, and iOS (iPhone). *U.S. Geological Survey Open-File Report.* (2010).
15. Underwood, A. J. *Experiments in Ecology, their Logical Design and Interpretation using Analysis of Variance*. (*Camb. Univ. Press*, 1997).
16. National Oceanic and Atmospheric Administration Coral Reef Watch. 50-km pixel satellite data, Available at: http://coralreefwatch.noaa.gov (date of access Nov 2016).
